# Unraveling the key drivers of bacterial progesterone degradation

**DOI:** 10.1128/mbio.01077-25

**Published:** 2025-05-30

**Authors:** Gabriel Hernández-Fernández, Juan Ibero, José L. García, Beatriz Galán

**Affiliations:** 1 Department of Biotechnology, Centro de Investigaciones Biológicas Margarita Salas (CSIC) 54446, Madrid, Community of Madrid, Spain; 2 Department of Biochemistry and Molecular Biology, Chemical Sciences Faculty, Complutense University of Madrid 683967, Madrid, Community of Madrid, Spain; University of Washington School of Medicine, Seattle, Washington, USA

**Keywords:** progesterone, endocrine disruptors, Baeyer-Villiger monooxygenases, luciferase-like monooxygenase, testosterone acetate

## Abstract

Progesterone (PROG) is one of the most ubiquitous sexual hormones found as a pollutant in soil and water systems. Despite the fact that PROG can be degraded by various bacterial species, the pathways leading to its complete oxic mineralization remain unknown. In this study, we investigated bacterial progesterone catabolism using the steroid-degrading bacterium *Caenibius tardaugens* as a model, in which we demonstrated its capacity to degrade progestogens. The transcriptomic analyses in the presence of PROG showed the overexpression of *EGO55_13845* and *EGO55_13860* genes, coding a Baeyer-Villiger monooxygenase (BVMO) and a luciferase-like monooxygenase (LLM), respectively. Both genes are located next to two regulatory proteins forming a small gene cluster (named *pdc*) that can be found in other steroid-degrading bacteria. Mutagenic analyses and gene complementation allowed ascertaining that *EGO55_13845* and *EGO55_13860* genes are involved in PROG degradation. To assess their enzymatic activities, both proteins were overexpressed in *Escherichia coli,* showing that they catalyze a Baeyer-Villiger monooxygenation of PROG, resulting in the production of testosterone acetate. They are also active on 1,2-dehydroprogesterone, an intermediate in PROG degradation, converting it into boldenone acetate. BVMO and LLM enzymes are functionally redundant, as each can replace the other in metabolizing PROG. The presence of two functionally redundant BVMO and LLM enzymes in *C. tardaugens* can be explained by their distinct substrate preferences. Our results settle for the first time the genetic and biochemical basis for explaining how PROG is recognized and channeled into the 9,10-seco degradation pathway in a PROG-degrading bacterium.

**IMPORTANCE:**

This study investigates for the first time the key steps in bacterial progesterone (PROG) degradation, revealing new insights into the process. The main stages of PROG degradation were examined in the bacterium *Caenibius tardaugens*. The conducted transcriptomic analysis allows identifying the progesterone degradation cluster *pdc,* which is also present in other related bacteria. We demonstrated that Baeyer-Villiger monooxygenase and luciferase-like monooxygenase encoded within the *pdc* cluster catalyze the PROG Baeyer-Villiger monooxygenation, producing testosterone acetate. The activity redundancy can be explained by the difference in substrate specificity of each enzyme.

## INTRODUCTION

Endocrine disruptors (EDCs) are natural and synthetic compounds that interfere with endocrine systems, causing adverse effects in humans and leading to the feminization or masculinization of freshwater wildlife exposed to polluted rivers (1–3). Among EDCs, natural estrogens (e.g., 17β-estradiol), androgens (e.g., testosterone [TES]), and progestogens (e.g., progesterone [PROG]) are the most widespread sex hormones found as pollutants in soil and water. These contaminants enter the environment through human, domestic, and farm animal excretions, as well as pharmaceutical waste (4, 5).

PROG is an endogenous 21-carbon steroid hormone synthesized from cholesterol in the corpus luteum of the ovaries and the placenta during pregnancy. It plays a crucial role in the female menstrual cycle, pregnancy, and embryogenesis in humans and other species (6). Due to its functional role, PROG is extensively used in oral contraceptives alongside the synthetic estrogen 17α-ethynylestradiol, as well as in hormone treatments to prevent miscarriage, hormone replacement therapy for menopausal women, and veterinary medicine (6). As a result, PROG is widely consumed, excreted through urine by humans, livestock, and other vertebrates, and subsequently released into the environment. Progestogens have been detected in wastewater treatment plants at concentrations ranging from 0.11 to 110 ng/L, and in surface water at levels between 0.82 and 1.1 ng/L. Their endocrine-disrupting effects on aquatic species have been extensively documented (7). Although progesterone undergoes biodegradation, it can still be detected in the effluents of both industrial and municipal wastewater and even in the receiving rivers (8, 9). In this sense, it has been documented that minor transformation of progestogens can create other classes of steroids with different biological activities like potent androgens (10–12).

Gut microbiota can convert glucocorticoids into progestins, via 21-dehydroxylation, in the presence of H_2_ gas (13). This is an example of cooperative metabolism dependent on the presence of certain bacteria such as *Eggerthella lenta* and *Gordonibacter pamelaceae*, which carry out the 21-dehydroxylation, and *Escherichia coli*, which provides H_2_. More specifically, PROG can be metabolized in the gut to 5α- and 5β-pregnanolone, 20-dihydroxyprogesterone, 11-desoxycorticosterone, and 17α-hydroxyprogesterone, compounds that have different targets and/or functions in the human body (14).

Microbial degradation can contribute to eliminating natural or synthetic steroid hormones from polluted systems, and the persistence and fate of some of these compounds have been studied previously (15, 16). Nevertheless, only a limited number of organisms can mineralize steroids (17–19). The pathways for the complete oxic mineralization of TES and 17β-estradiol (E2) have been studied in detail in several bacteria (20–24). However, although bacteria capable of degrading PROG have been described (25–27), there is limited information on the genes involved in the first steps of its mineralization, and only a few biochemical studies have been published. Liu et al. studied the aerobic degradation of PROG and norgestrel, detecting 4-androstene-3,17-dione (AD), androsta-1,4-diene-3,17-dione (ADD), 17β-boldenone, testosterone, 6,7-dehydroprogesterone, and 1,2-dehydroprogesterone (1,2-dPROG) as intermediates in the culture medium (28). They proposed that PROG undergoes different transformations ending up in ADD or 17β-boldenone, which are finally mineralized. Horinouchi et al. suggested that *Comamonas testosteroni* degrades PROG through the well-known 9,10-seco pathway used as well in the bacterial mineralization of sterols, bile acids, and androgens, but the genes and the enzymes that channel PROG into this degradative pathway were not described (20).

It is known that some fungal species can degrade and cleave the side chain of PROG, transforming it into potent androgens like testololactone, which has anticancer activity, through several steps mediated by Baeyer-Villiger monooxygenases (BVMOs). However, the activity has been demonstrated only through whole cell biotransformations, and the enzymes responsible for these biochemical steps have not been isolated and identified so far (29, 30). Based on the identification of the biotransformation intermediates, it has been postulated that during side chain degradation of PROG, oxygen is typically introduced between C17 and C20 to form 17-acetate, followed by cleavage of the side chain, leading to the formation of androgens with a C19 skeleton, particularly TES. TES is then oxidized to form AD, a C19 compound with a 3-keto group. The final and critical step is the conversion of AD to testololactone. In this sense, BVMOs catalyze the insertion of an oxygen atom into the 3-keto group of AD, which results in the formation of a C19 lactone ring in testololactone (29).

The activity of a bacterial steroid monooxygenase, mediated by BVMOs, introducing an oxygen atom between the C17 and C20 carbons of PROG, was first demonstrated in the gram-positive actinomycete *Rhodococcus rhodochrous* (formerly *Nocardia corallina*) by Miyamoto et al. (31). The gene encoding this enzyme was cloned by Morii et al. (32), and the protein was structurally characterized by Franceschini et al. (33). The properties of the enzyme differed considerably from those of the corresponding fungal monooxygenases because it catalyzed oxidative esterification of PROG to testosterone acetate (TES-Ac) as well as its 11(α/β)-hydroxy derivatives, but the lactonization of AD was not observed. Although these studies confirm the presence of a PROG-transforming enzyme in this microorganism, they did not describe the PROG catabolic pathway. Other BMVOs from *Xanthobacter*, *Comamonas,* and *Rhodococcus* have been cloned and assayed by Van Beilen et al. (34), but none of them showed activity on PROG.

**Fig 1:**
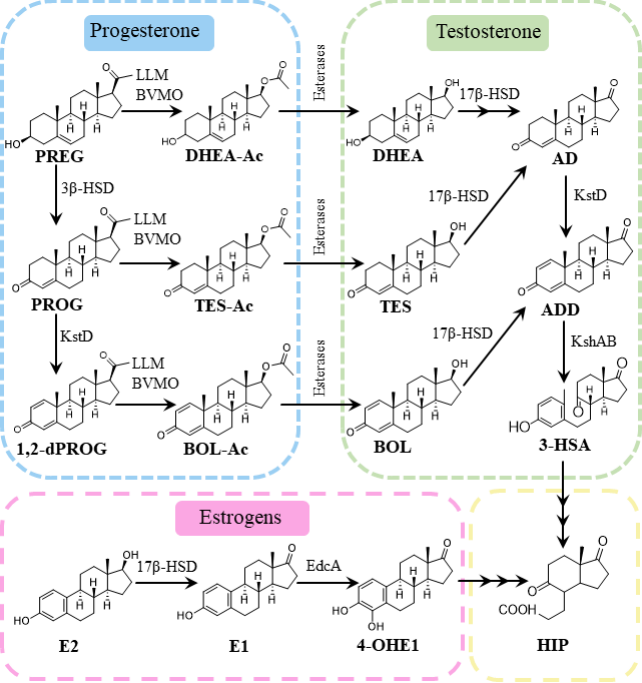
Scheme of all the steroid-degrading routes described in *C. tardaugens*. The progesterone degradation pathway described in this work is highlighted in blue. The testosterone degradation pathway is marked in green while the estrogen degradation pathway is colored in pink, and both are described by Ibero et al. (20, 21). PREG, pregnenolone; DHEA-Ac, androstenediol acetate; BOL-Ac, boldenone acetate; DHEA, dehydroepiandrosterone; BOL, boldenone; 3-HSA, 3-hydroxy-9,10-secoandrosta-1,3,5(10)-triene-9,17-dione; E1, estrone; 4-OHE1, 4-hydroxyestrone; HIP, 3α-H-4α(3′-propanoate)-7α-β-methylhexahydro-1,5-indanedione; LLM, luciferase-like monooxygenase; 3β-HSD, 3-hydroxysteroid dehydrogenase; 17β-Hsd, 17β-hydroxysteroid dehydrogenase; KstD, 3-ketosteroid-Δ1-dehydrogenase; KshAB, 3-ketosteroid 9α-hydroxylase; EdcA, estrone 4-hydroxylase.

In this work, we have studied the PROG catabolism using as a model the gram-negative alphaproteobacterium *Novosphingobium tardaugens* NBRC 16725 (described as strain ARI-1) and now renamed as *Caenibius tardaugens* (35). This bacterium was isolated at a sewage treatment plant in Tokyo, Japan, due to its ability to use estrogens as the only carbon and energy source (36). Its curated genome is now available (37), and it was used as a model to elucidate the E2 and TES degradation pathways (21, 22) (Fig. 1). Here, for the first time, we identify the genes and characterize the enzymes involved in the PROG degradation pathway in a bacterium capable of degrading a large variety of endocrine disruptors.

## RESULTS

### *C. tardaugens* grows on a wide range of steroids

**Fig 2:**
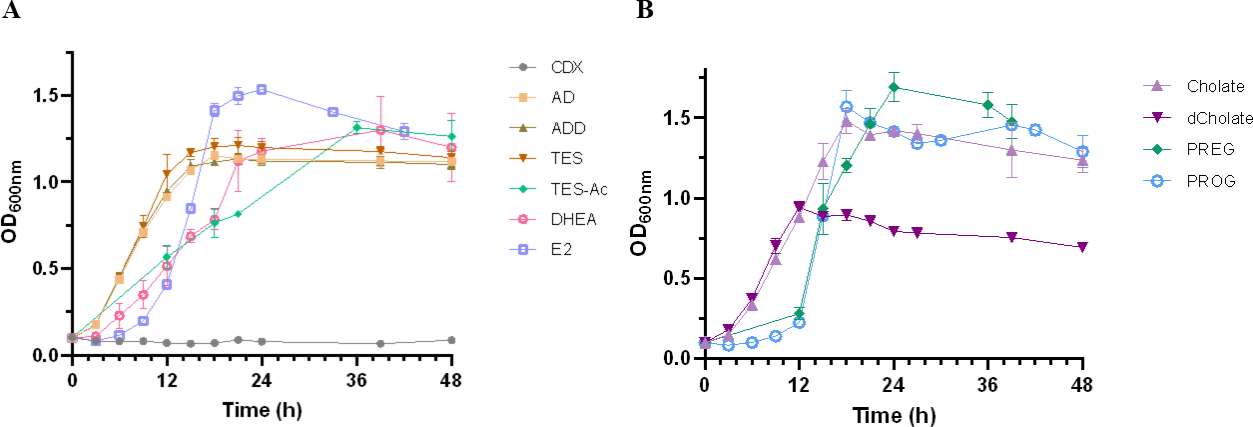
(**A**) Growth curves of *C. tardaugens* NBRC 16725 on different C19 steroids. The values represented are the mean of three independent biological replicates. (**B**) Growth curves of *C. tardaugens* NBRC 16725 on different steroids with side chain. Cholate, sodium cholate. The values represented are the mean of three independent biological replicates.

Figure 2 shows that *C. tardaugens* is able to use different steroids as sole carbon and energy sources. *C. tardaugens* efficiently uses different C18 and C19 steroids without side chains such as E2, estrone, and estriol (22), AD, ADD, TES, dehydroepiandrosterone (DHEA), TES-Ac, and testosterone cypionate (TES-Cyp) (Fig. 2A), as well as steroids with different side chain lengths such as deoxycholate (dCholate), cholate, pregnenolone (PREG), and PROG (Fig. 2B). Cyclodextrins (CDX) used to solubilize steroids are not metabolized by this strain. These results confirm that *C. tardaugens* is the only tested gram-negative bacterium capable of degrading a wide variety of steroid substrates. This ability suggests that it is equipped with a great arsenal of metabolic pathways and makes it an excellent model for studies on the biodegradation of steroidal endocrine disruptors. To date, we have identified and characterized the metabolic pathways to degrade estrogens (C18 steroids) and androgens (C19 steroids) in this strain (21, 22), but the key drivers responsible for degrading steroids containing side chains at C17, such as progesterone, have not been described yet. Based on this, we decided to investigate the pathway responsible for PROG metabolism. Although PROG is one of the central EDC pollutants, to date, the microbial genes involved in its degradation have not been identified and/or described.

### A transcriptomic analysis of *C. tardaugens* grown on progesterone reveals the presence of the *pdc* cluster

To identify the genes involved in the degradation of PROG, we performed RNA-seq analysis. Total RNA was extracted from *C. tardaugens* cultured in PROG and in TES, as the control condition. The differential expression analysis of these data yielded 36 differentially expressed genes (DEGs) out of the 4,080 genes encoded in the genome (Table S1). Among the 36 DEGs, 33 were upregulated with a fold change (FC) > 2 and only 3 were downregulated in PROG with FC < −2 (Fig. S1), suggesting that there are few differences between the TES and PROG degradation pathways, most probably because both might converge at a certain point. Previous transcriptomic data of this strain allowed us to describe in detail the TES degradation cluster (*SD* cluster) and the E2 degradation cluster (*edc* cluster) of *C. tardaugens* through differential expression analysis in the presence of TES or E2 vs pyruvate (20, 21).

**Fig 3:**
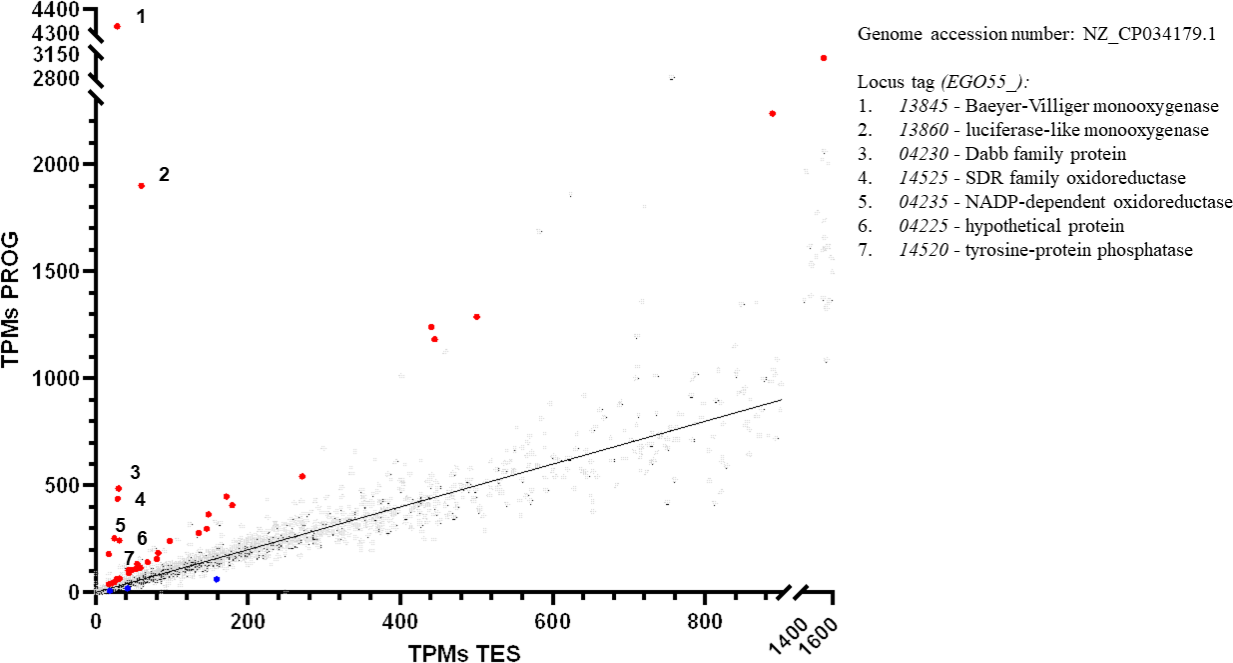
Distribution of the DEGs according to their expression in progesterone or testosterone expressed in TPMs. Red dots represent DEGs with FC > 2 and false discovery rate (FDR) < 0.05, while blue dots represent DEGs with FC < −2 and FDR < 0.05. Gray dots correspond to genes showing 2 > FC > −2. The seven DEGs more overexpressed are marked from 1 to 7 with their locus tag. SDR: short-chain dehydrogenase/reductase.

Figure 3 presents a comparison of the transcripts per kilobase million (TPMs) of each gene expressed in PROG vs TES, highlighting the most upregulated genes that may play a role in PROG metabolism. The highest level of upregulation FC > 5 was observed in seven genes grouped in three different clusters: *EGO55_04225–04235*, *EGO55_13845–EGO55_13860,* and *EGO55_14520–EGO55_14525*. The *EGO55_04225–04235* cluster encodes a hypothetical protein, a Dabb family protein, and a NADP-dependent oxidoreductase, showing an FC of 9, 15, and 11, respectively (Table S1). *EGO55_14520* encodes a tyrosine phosphatase with an FC of 8, while *EGO55_14525* encodes an short-chain dehydrogenase/reductase (SDR) family oxidoreductase with an FC of 15 (Table S1). Notably, the highest FC values were found for the cluster containing the *EGO55_13845* and *EGO55_13860* genes, encoding a BVMO and a luciferase-like monooxygenase (LLM) with values of 139 and 32, respectively. Within this cluster, two additional genes, *EGO55_13850* and *EGO55_13855*, encoding transcriptional regulators, are not induced in PROG, displaying FC values of −1 and 3, respectively (Table S1). This cluster is 12.8 kb distant from the *SD* cluster (TES degradation) (20) and 51.4 kb distant from the *edc* cluster (E2 degradation) (21), and therefore, in principle, the detected cluster cannot be directly associated with these degradation pathways. Considering the high level of upregulation of the *EGO55_13845–EGO55_13860* cluster, we have named the *pdc* cluster that stands for PROG degradation cluster and selected it for further studies.

### Annotation of the *pdc* cluster

**Fig 4:**
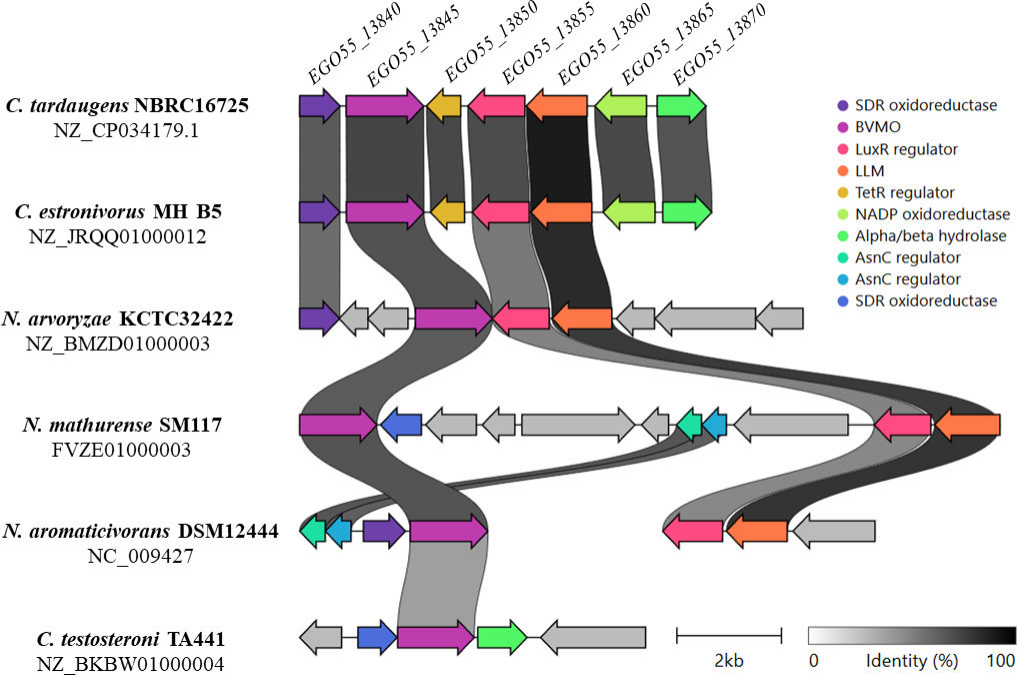
Gene organization of the *pdc* cluster in *C. tardaugens* compared to other related bacteria.

The *pdc* cluster of *C. tardaugens* spans 4.6 kb and comprises four genes (Fig. 4). *EGO55_13845* encodes a BVMO that contains a transmembrane domain and a non-cytoplasmic domain consisting of a NAD(P)-binding Rossmann-like domain and an FAD-binding domain. Baeyer-Villiger monooxygenases catalyze the oxidation of a ketone group to an ester group using NAD(P)H and molecular oxygen, with the release of a water molecule (38). BVMOs can be classified into two main groups depending on whether they bind FAD or FMN as cofactors (38, 39). Type I BVMOs have two Rossman folds enabling the protein to bind FAD and NAD(P)H, so that a single protein can perform the oxidation and reduction activities (33, 40). On the other hand, type II BVMOs have the oxygenating and the reducing components in two different proteins: the reducing component binds NADH and reduces FMN to FMNH_2_, and the oxygenating component contains an eight-stranded α/β-barrel to bind FMNH_2_ and molecular oxygen to perform the oxidation (41). The Baeyer-Villiger reaction has been described in several BVMOs from fungi and other bacteria due to their interest as a high regio-, stereo-, and enantioselective biocatalyst over different aliphatic and aryl ketones and steroids. BVMOs catalyze the insertion of an oxygen atom into ketone substrates to generate lactones or esters.

*EGO55_13845* encodes a type I BMVO with a 30.4% amino acid sequence identity to that of *R. rhodochrous* that transforms PROG into TES-Ac *in vitro* (31–34). A large number of putative type I BVMOs are encoded in the *C. tardaugens* genome, sharing varying degrees of similarity. One of the most intriguing is the BVMO encoded by the *EGO55_06865* gene, located 1.4 Mbp away from the *pdc* cluster. This BVMO shares a 47.6% amino acid sequence identity with the BVMO from *R. rhodochrous* and a 53.0% amino acid sequence identity with the BVMO encoded by *EGO55_13845*. However, the transcriptomic analysis showed that the *EGO55_06865* gene is not differentially expressed. Moreover, the TPMs for *EGO55_06865* were around 50, while those for *EGO55_13845* were above 4,000 (Table S1), suggesting that *EGO55_06865* is likely not involved in PROG metabolism, or at least cannot be induced under the culture conditions used in this study. BVMOs highly similar to EGO55_13845 can be found in other well-known steroid-degrading bacteria, such as *Croceicoccus estronivorus* MH-B5, *Novosphingobium aromaticivorans* DSM 12444, and *C. testosteroni* TA441, sharing 79%, 71%, and 47% protein sequence identity, respectively (Fig. 4). Interestingly, the BVMO (CTTA_RS14345) from *C. testosteroni* TA441 shares 47% identity with EGO55_13845 but a 55% identity with EGO55_06865. Two putative regulatory genes are located in the *pdc* cluster. *EGO55_13850* gene encodes a TetR/AcrR family transcriptional regulator, whereas the *EGO55_13855* gene encodes a helix-turn-helix transcriptional regulator belonging to the LuxR family. The *EGO55_13850* gene is slightly downregulated, showing an FC of −1 (Table S1). This might suggest that the encoded regulator may operate as a repressor of the *pdc* cluster, according to the main function of the TetR-like regulator (42). In contrast, the expression of the *EGO55_13855* gene was upregulated in the presence of PROG with an FC of 3 (Table S1), suggesting that the encoded regulator might act as an activator. Curiously, the TES degradation pathway in *C. testosteroni* TA441 is induced by TesR, a similar LuxR-type transcriptional regulator (43). Both regulators have homologous proteins in other bacteria with an amino acid sequence identity ranging from 55 to 78% (Fig. 4). However, no homologous gene to *EGO55_13850* was found in the genome of *Novosphingobium arvoryzae* KCTC 32422 having a similar *pdc* cluster to that of *C. tardaugens* (Fig. 4).

Finally, the *EGO55_13860* gene encodes an LLM containing one luciferase-like domain characterized by displaying a triose-phosphate isomerase (TIM)-barrel folding, which is the typical domain present in type II BVMOs (41). There are only a few biochemical studies involving this kind of BVMOs, probably because the catalysis involves a two-protein complex, one monooxygenase and one reductase. The LLM EGO55_13860 present in the *pdc* cluster might be the oxygenation component in the Baeyer-Villiger reaction requiring a flavin reductase. The *EGO55_13865* gene located next to *EGO55_13860* encoded a protein annotated as a NADP-dependent oxidoreductase, and according to the transcriptomic analysis, its expression remains below 30 TPMs in the presence of PROG with an FC around −1. Therefore, we cannot ascribe this reductase to the second component providing the flavin reductase required for the LLM activity. There is only one flavin reductase within the *C. tardaugens* genome encoded by the *EGO55_18005* gene located distant from the *pdc* cluster. Its expression in the transcriptome is around 100 and 170 TPMs in PROG and TES conditions, respectively, with an FC of −1 (Table S1). These values are far from the TPMs > 1,700 and FC > 30 observed for *EGO55_13860*, but the low expression levels might be enough for its reduction activity, avoiding excessive metabolic burden in the bacterial cell. The LLM EGO55_13860 has no other homologous proteins in the *C. tardaugens* genome. However, LLM homologs can be found in other steroid-degrading bacteria, such as MB02_15840, IE113_RS08665, and Saro_3594 from *C. estronivorus* MH-B5, *N. arvoryzae* KCTC 32422, and *N. aromaticivorans* DSM 12444, sharing 92%, 87%, and 88% of protein sequence identity, respectively (Fig. 4). No LLM EGO55_13860 homologous proteins were found in the PROG-degrading bacterium *C. testosteroni* TA441 (Fig. 4). However, it is worth mentioning that *Mycolicibacterium smegmatis* is able to use C19 steroids when the ADD+ cluster is derepressed (44). Interestingly, this ADD+ cluster contains an LLM protein encoded by *MSMEG_4862,* having 28% aa identity with LLM EGO55_13860, which can explain why *M. smegmatis* can use PROG as substrate after activation of the ADD+ cluster (personal communication).

### BVMO and LLM are key enzymes in PROG degradation

**Fig 5:**
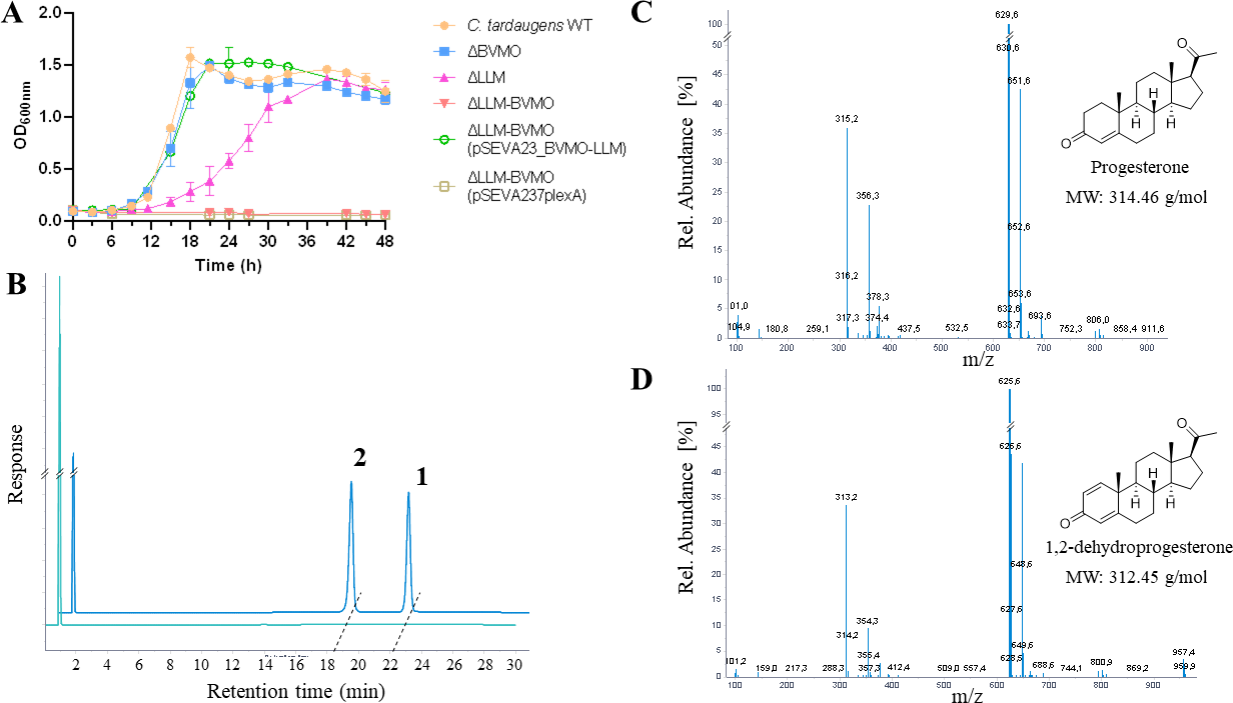
(**A**) Growth curve of *C. tardaugens* WT, ΔBVMO, ΔLLM, ΔLLM-BVMO, ΔLLM-BVMO (pSEVA23_BVMO-LLM), and ΔLLM-BVMO (pSEVA237plexA) strains on 1.71 mM PROG. (**B**) High-performance liquid chromatograph with mass spectrometry (HPLC-MS) analysis of the organic phase extracted from *C. tardaugens* WT (turquoise) and ΔLLM strain (blue) culture grown in progesterone for 24 h. Peak 1 corresponds to progesterone, and peak 2 corresponds to 1,2-dPROG. (**C**) Mass spectra of peak 1, corresponding to PROG. (**D**) Mass spectra of peak 2, corresponding to 1,2-dPROG.

The *EGO55_13845* and *EGO55_13860* genes encoding the BVMO and LLM enzymes, respectively, were deleted in *C. tardaugens* in order to prove their role in the PROG degradation pathway. Through bacterial conjugation and using the suicidal plasmid pk18msg (Table 1), harboring the 700 bp upstream and downstream regions of *EGO55_13845* and *EGO55_13860*, two new strains were obtained: *C. tardaugens* ΔBVMO and *C. tardaugens* ΔLLM. Both mutants showed a similar growth to that of the wild-type (WT) strain when growing in nutrient broth (NB), suggesting that *EGO55_13845* and *EGO55_13860* are not essential genes for *C. tardaugens* basal metabolism. However, unexpectedly, when both strains were cultured in the presence of 1.71 mM PROG as sole carbon and energy source, both mutants were able to grow, although a longer lag phase was observed in the *C. tardaugens* ΔLLM mutant (Fig. 5A). These results might suggest that both enzymes are not involved in the metabolism of PROG, but considering that both enzymes could display similar activities, they can provide an enzymatic redundancy. Even more, considering that BVMO is very similar to that EGO55_06865, this enzyme might take its function.

**TABLE 1:** Bacterial strains, plasmids, and primers used in this study

Strain, plasmid, or primer	Genotype and/or description	Reference or sequence
Strains		
*C. tardaugens*		
NBRC 16725	Wild-type strain	(27)
ΔBVMO	NBRC 16725 *ΔEGO55_13845*	This work
ΔLLM	NBRC 16725 *ΔEGO55_13860*	This work
ΔLLM-BVMO	NBRC 16725 *ΔEGO55_13845–13860*	This work
ΔLLM-BVMO (pSEVA23_BVMO-LLM)	NBRC 16725 *ΔEGO55_13845–13860* with pSEVA23pLexA-EGO55_13845–13860	This work
ΔLLM-BVMO (pSEVA237plexA)	NBRC 16725 *ΔEGO55_13845–13860* with pSEVA237pLexA	This work
*E. coli*		
HB101	*supE44, ara14, galK2, leuB, lacY1,* Δ(*gpt-proA*)62, *rps*L20, *xyl-5, mtl-1*, *recA13*, Δ(*mcrC-mrr*), *hsdS20,* (rB^−^, mB^−^), Sm^R^	(45)
DH10B	F^−^, *mcrA*, Δ(*mrr, hsdRMS-mcrBC*), φ80d*lacZ*ΔM15, *ΔlacX74*, *deoR*, *recA1*, *araD139*, Δ(*ara-leu*)7697, *galU*, *galK*, λ^−^, *rpsL*, *endA1*, *nupG*	Invitrogen, UK
DH10B (pK18EGO55_13845)	DH10B with pK18EGO55_13845	This work
DH10B (pK18EGO55_13860)	DH10B with pK18EGO55_13860	This work
DH10B (pSEVA23_BVMO-LLM)	DH10B with pSEVA23pLexA-*EGO55_13845–13860*	This work
DH10B (pSEVA237plexA)	DH10B pSEVA237pLexA	This work
BL21(DE3)	F^−^, *ompT*, *hsdSB* (rB^−^ mB^−^) *gal*, *dcm* λDE3 (harboring gene 1 of the RNA polymerase from phage T7 under the *P_lacUV5_* promoter)	(46)
BL21(DE3) (pET-29(+)BVMO)	BL21(DE3) with pET-29(+) *EGO55_13845*	This work
BL21(DE3) (pET-29(+)LLM)	BL21(DE3) with pET-29(+) *EGO55_13860*	This work
BL21(DE3) [pET-29a(+)]	BL21(DE3) with pET-29(+)	This work
Plasmids		
pRK600	Cm^R^, ColE1 *oriV,* RP4 *oriT*; helper plasmid necessary for triparental mattings	(47)
pK18msg	Km^R^, ColE *oriV*, Mob^+^, *lacZα*, *sacB*; vector for gene deletion via homologous recombination	(46)
pK18EGO55_13845	pK18mobsacB derivative containing the upstream and downstream fragments of *EGO55_13845* cloned with *Bsa*I for directed mutagenesis	This work
pK18EGO55_13860	pK18mobsacB derivative containing the upstream and downstream fragments of EGO55_13860 cloned with *Bsa*I for directed mutagenesis	This work
pSEVA237plexA	Km^R^, pBBR1 *oriV*, constitutive expression of *gfp* gene under the control of the *P_lexA_* promoter	(48)
pSEVA23_BVMO-LLM	pSEVA237pLexA where the *gfp* gene was replaced by *EGO55_13845–13860* between *XbaI* and *SpeI*	This work
pET-29a(+)	Cloning and expression vector, Km^R^, ColE1 *oriV*, T7 promoter	Novagen, Germany
pET-BVMO	pET-29 containing the *EGO55_13845* gene	This work
pET-LLM	pET-29 containing the *EGO55_13860* gene	This work
Primers		
EGO55_13845UpFw_BsaI	Amplification of the fragment upstream *EGO55_13845* (*Bsa*I)	5′-CACATGGTCTCTTCTTGTCGATATCACCGATCA-3′
EGO55_13845UpRv_BsaI		5′-CACATGGTCTCGTCATCCCCGTTACTGTTCTGAT-3′
EGO55_13845DwFw_BsaI	Amplification of the fragment downstream *EGO55_13845* (*Bsa*I)	5′-CACATGGTCTCGATGACTTGTCTGATATCTGTTGGA-3′
EGO55_13845DwRv_BsaI		5′-CACATGGTCTCGTTCCCACTTATGACACGAAAGC-3′
EGO55_13860UpFw_BsaI	Amplification of the fragment upstream *EGO55_13860* (*Bsa*I)	5′-CACATGGTCTCTTCTTGGTCGGCAATGATC-3′
EGO55_13860UpRv_BsaI		5′-ACATGGTCTCGTCATCATGCCCTGATTTATGTC-3′
EGO55_13860DwFw_BsaI	Amplification of the fragment downstream *EGO55_13860* (*Bsa*I)	5′-CACATGGTCTCGATGACCTTCTCTCCCGAGCT-3′
EGO55_13860DwRv_BsaI		5′-CACATGGTCTCGTTCCCGAACAACTCTGCCTAT-3′
EGO55_13845FwXbaI	Amplification of *EGO55_13845* (*Xba*I-*Sph*I)	5′-ATATTCTAGAAAGGAGGTGAATATGTCCTCTGTGAATAACGGGT-3′
EGO55_13845RvSphI		5′-ATATGCATGCCTAGCTGATGACGTAATCGG-3′
EGO55_13860FwHindIII	Amplification of *EGO55_13860* (*Hin*dII*I-Spe*I)	5′-ATATAAGCTTAAGGAGGTGAATATGAAGTTTTCCATCATTTATGAA-3′
EGO55_13860RvSpeI		5′-ATATACTAGTTCAGCCAGCCACAGTTTCAA-3′
GibEGO55_13845Fw	Amplification of *EGO55_13845* and cloning by Gibson assembly	5′-TAAGAAGGAGATATACATATGTCCTCTGTGAATAACG-3′
GibEGO55_13845Rv		5′-GTGGTGGTGGTGGTGGTGGCTGATGACGTAATCG-3′
GibEGO55_13860Fw	Amplification of *EGO55_13860* and cloning by Gibson assembly	5′-TAAGAAGGAGATATACATATGAAGTTTTCCATCATTTA-3′
GibEGO55_13860Rv		5′-GTGGTGGTGGTGGTGGTGGCCAGCCACAGTTT-3′

To determine a possible redundancy in the function of both enzymes, we constructed a double mutant performing the mutation of BVMO in the *C. tardaugens* ΔLLM strain, yielding the *C. tardaugens* ΔLLM-BVMO strain. Supporting the redundancy hypothesis, the double mutant did not grow when cultured with 1.71 mM PROG as sole carbon and energy source (Fig. 5A). To further confirm that BVMO and LLM are required to metabolize PROG, the *C. tardaugens* ΔLLM-BVMO strain was transformed with plasmid pSEVA23_BVMO-LLM, harboring the *EGO55_13845* and *EGO55_13860* genes under the expression of *P_lexA_* promoter, thus resulting in the new strain *C. tardaugens* ΔLLM-BVMO (pSEVA23_BVMO-LLM). As control, *C. tardaugens* ΔLLM-BVMO mutant was transformed with the pSEVA237plexA empty vector, yielding the *C. tardaugens* ΔLLM-BVMO (pSEVA237plexA) strain. When both strains were cultured with PROG as the sole carbon and energy source, only the complemented *C. tardaugens* ΔLLM-BVMO (pSEVA23_BVMO-LLM) strain recovered the ability to grow (Fig. 5A), demonstrating that the double mutant has no additional mutations that can impair its growth in PROG.

Using the *C. tardaugens* ΔLLM and *C. tardaugens* ΔBVMO mutants, we searched for the presence of intermediate compounds from PROG degradation in the extracellular medium from their cultures in the presence of 1.71 mM PROG. Samples were taken every 3 h, and total steroids were extracted with chloroform to be analyzed by high-performance liquid chromatograph with mass spectrometry (HPLC-MS). After 24 h of culture, PROG is still detected in the culture medium of *C. tardaugens* ΔLLM mutant when compared to the wild-type strain, but a new peak was detected in the chromatograms (Fig. 5B). The fragmentation pattern of this peak indicated a molecular weight of 313 [M+H]^+^, which might correspond to a dehydrogenated version of PROG. This finding was confirmed by comparing this peak with the peak of commercial 1,2-dPROG (Fig. 5C). In cultures of *C. tardaugens* ΔLLM, most of the PROG was transformed into 1,2-dPROG after 30 h and no substrate was observed after 39 h (Fig. S2). 1,2-dPROG was also detected after 18 h in the supernatants of cultures of the *C. tardaugens* ΔBVMO strain, even though this mutant did not show a longer lag phase when compared to the wild-type strain. However, in this case, all 1,2-dPROG and PROG were consumed after 24 h of cultivation (Fig. S3).

In the case of the *C. tardaugens* ΔLLM-BVMO strain, cells were cultured in a medium containing glucose as carbon and energy source and 1.71 mM PROG to detect possible intermediates. As expected, 1,2-dPROG was detected in the medium, and it remained stable throughout 96 h of cultivation (Fig. S4), demonstrating that this mutant is capable of transforming PROG into the 1,2-dPROG intermediate, but is not able to use PROG as a carbon and energy source.

### BVMO and LLM catalyze the transformation of PROG into TES-Ac

To determine the precise role of BVMO and LLM enzymes in PROG catabolism, we performed *in vitro* biochemical assays with BVMO and LLM using PROG as substrate.

**Fig 6:**
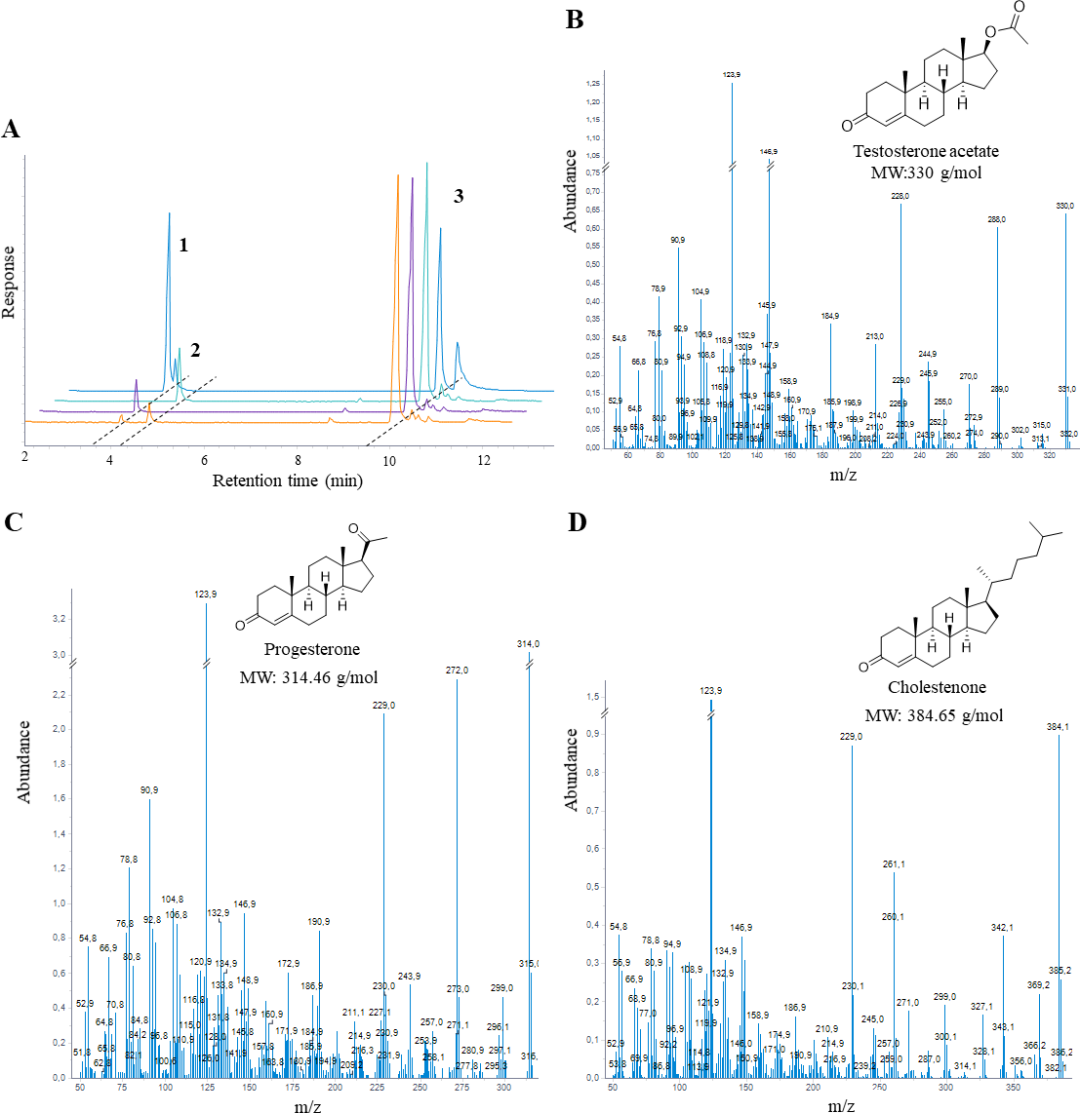
(**A**) Gas chromatography-mass spectrometry (GC-MS) analysis of the organic phase extracted from the enzymatic reaction of progesterone with crude extracts from *E. coli* BL21(DE3) (pET-29(+)BVMO) (orange), *E. coli* BL21(DE3) (pET-29(+)LLM) (purple) and *E. coli* BL21(DE3) [pET29(+)] (turquoise). Commercial testosterone acetate is represented in blue. Peaks correspond to (1) TES-Ac, (2) PROG, and (3) cholestenone (ISTD). (**B**) Mass fragmentation pattern of peak 1 in GC-MS. (**C**) Mass fragmentation pattern of peak 2 in GC-MS. (**D**) Mass fragmentation pattern of peak 3 in GC-MS.

We initially conducted enzymatic assays using crude protein extracts from *C. tardaugens* WT and mutant strains cultured in PROG, but we were unable to detect the formation of TES-Ac (Fig. S5), most probably due to the low expression of the enzymes. Therefore, to further characterize their activities, we overexpressed the enzymes in a heterologous system. To this aim, the genes encoding BVMO and LLM were cloned in the pET29a(+) expression vector, yielding pET-29(+)BVMO and pET-29(+)LLM, respectively. Both plasmids were transformed in *E. coli* BL21(DE3), and the enzymes were overexpressed (Fig. S6). Crude protein extracts were used to perform an *in vitro* enzymatic assay using PROG and NAD(P)H as substrates (see Materials and Methods). After 30 min of reaction, the steroids were extracted and analyzed by gas chromatography-mass spectrometry (GC-MS) and compared to the control with the empty plasmid pET29(+). Both BVMO and LLM enzymatic assays deliver a new peak when analyzed by GC-MS (Fig. 6A). According to its fragmentation pattern and the NIST Mass Spectral Database, it was identified as TES-Ac (Fig. 6B). This result confirmed that *pdc*-encoded BVMO and LLM are able to transform PROG into TES-Ac through a Baeyer-Villiger oxidation.

### BVMO and LLM exhibit differential activity on PROG derivatives

We have demonstrated that the BVMO and LLM enzymes have an important role in PROG catabolism in *C. tardaugens*. However, they exhibit redundancy in their enzymatic reactions, as both monooxygenases catalyse a Baeyer-Villiger oxidation of PROG, leading to the formation of TES-Ac. To explore new substrates for both monooxygenases and evaluate the different activities that may explain their essential role in the PROG degradation pathway, an enzymatic assay using progesterone-related steroids was conducted. Crude extracts from *E. coli* BL21(DE3) containing pET-29(+)BVMO and pET-29(+)LLM plasmids were obtained. The enzymatic activity was analyzed on four steroids: PROG as the native substrate, 1,2-dPROG as the intermediate detected in PROG degradation, 21-hydroxyprogesterone (21-OHPROG) as a steroid having a modification on the side chain, and PREG as the reduced and isomerized PROG derivative. The activity of the BVMO was assessed by measuring the decrease in absorbance of 0.2 mM NADPH at 340 nm when 0.5 mM of substrate was added. To assess the basal consumption of NADPH of the crude extract, NADPH was added with no substrate. The BVMO EGO55_13845 exhibited the highest specific activity on PROG and PREG (70.4 and 68.5 U/mg, respectively) (Table 2), indicating that, among all the substrates tested, PROG and PREG are preferred by the enzyme, with no significant difference between them. Nevertheless, when 1,2-dPROG was tested as substrate, the specific activity dropped to 41 U/mg, suggesting that the new double bond between C1 and C2 may hinder the proper positioning of this steroid in the substrate binding site. The lowest specific activity was observed in the presence of 21-OHPROG (8.7 U/mg) (Table 2), which is an expected result due to the fact that the hydroxyl group next to the ketone group may cause a steric hindrance in the protein’s active site. The specific activity of the LLM enzyme was defined as the nanomole of substrate consumed per minute per milligram of protein, as measuring NADH consumption would be an indirect method due to the involvement of a reducing partner in the reaction. The reaction was initiated by adding 5 mM NADH, and samples were collected after 5 and 30 min for analysis via HPLC-MS. LLM exhibited similar specific activity with PROG, PREG, and 1,2-dPROG (10.1, 12.5, and 14.7 U/mg, respectively) (Table 2), with 1,2-dPROG showing a slightly higher value. As observed in the BVMO assays, the specific activity of LLM on 21-OHPROG was the lowest, reaching only 6.4 U/mg. Additionally, after 30 min of incubation, PROG and PREG remained detectable in the sample, while 1,2-dPROG and 21-OHPROG were no longer detected (Fig. S7 to S9). Although these enzymatic assays with both monooxygenases were performed using crude extracts and cannot be directly compared, it is evident that each enzyme exhibits distinct substrate activities, which may explain their specific biological roles within the *pdc* cluster. While BVMO preferred substrates are PROG and PREG, LLM exhibits similar activity on PROG, PREG, and 1,2-dPROG, suggesting that the substrate binding domain of LLM can accommodate steroids with structural modifications in ring A. Both monooxygenases share the common trait that any modification in the side chain of PROG (e.g., hydroxylation) hinders their activity. In order to precisely identify the product obtained in the enzymatic assays, the samples were analyzed by GC-MS. When 1,2-dPROG was used as substrate, both monooxygenases produced boldenone acetate (BOL-Ac) (Fig. S7). PREG was transformed into androstenediol 17-acetate (Fig. S8), and despite a low specific activity, 21-OHPROG was oxidized into testosterone 2-hydroxyacetate (Fig. S9).

**TABLE 2:** Specific activity over different substrates of crude extracts containing BVMO EGO55_13845 or LLM EGO55_13860 from *C. tardaugens*

Enzyme	Substrate	Specific activity (U/mg)^*a*^
BVMO EGO55_13845	Progesterone	70.4
	21-Hydroxyprogesterone	8.7
	1,2-Dehydroprogesterone	41
	Pregnenolone	68.5
LLM EGO55_13860	Progesterone	10.1
	21-Hydroxyprogesterone	6.4
	1,2-Dehydroprogesterone	14.7
	Pregnenolone	12.5

^*a*^ For BVMO, specific activity is defined as the amount of NADPH (nmol) consumed per minute per milligram of protein. For LLM, specific activity is defined as the number of nmol of substrate consumed per minute per milligram of protein.

These data suggest that BVMO and LLM are induced in order to catalyze a Baeyer-Villiger oxidation on PROG to produce TES-Ac *in vivo*. However, when PROG enters the cell, a fraction of PROG can be dehydrogenated, before being transformed into TES-Ac, by a 3-ketosteroid-Δ1-dehydrogenase (KstD) to yield 1,2-dPROG, requiring the involvement of enzymes capable of performing monooxygenation on both substrates, PROG and 1,2-dPROG. We suggest that in *C. tardaugens,* 1,2-dPROG monooxygenation activity might be supplied mainly by LLM EGO55_13860. This hypothesis is supported by the longer lag phase observed in the ΔLLM mutant strain growing on PROG (Fig. 5A), where the accumulation of 1,2-dPROG is observed up to 39 h of growth (Fig. S2).

### *C. tardaugens* PROG metabolism is linked to TES metabolism

**Fig 7:**
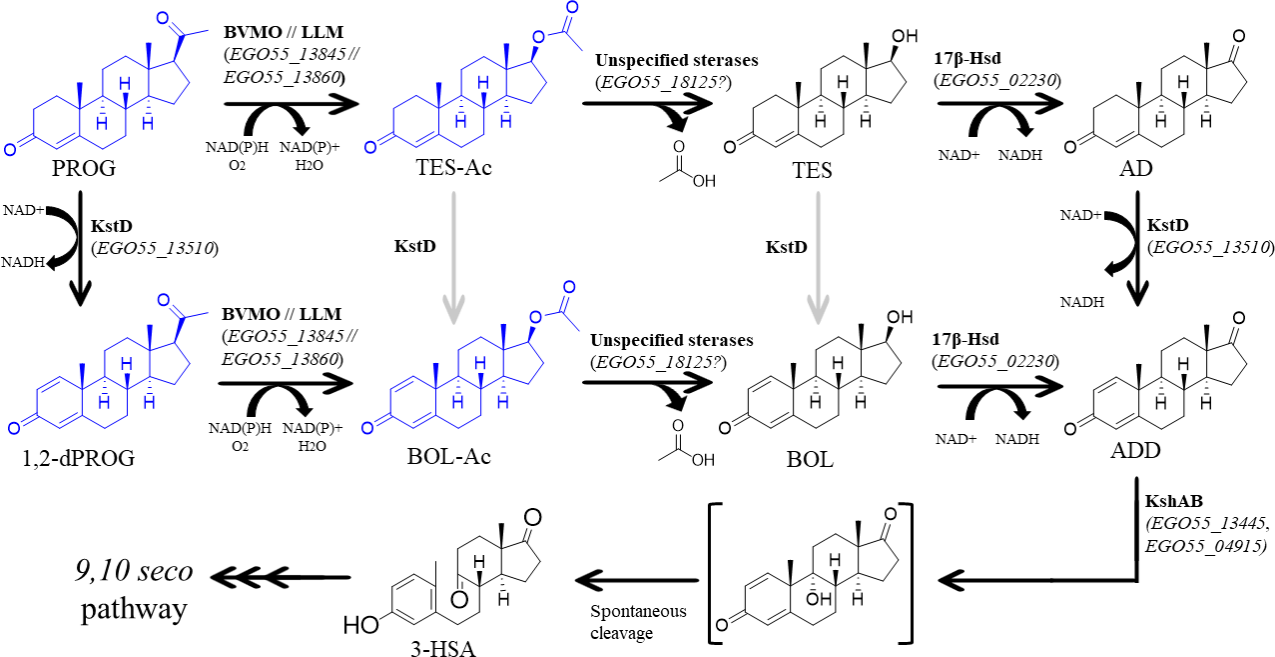
Proposed progesterone degradation pathway in *C. tardaugens* NBRC 16725. Enzymes catalyzing the reaction are marked in bold, and their proposed encoding genes are specified in brackets. The compounds detected in this work are colored in blue. Compound names are indicated with an abbreviation: 3-HSA, 3-hydroxy-9,10-secoandrosta-1,3,5(10)-triene-9,17-dione. Enzyme names are BVMO; LLM; 17β-Hsd, 17β-hydroxysteroid dehydrogenase; KshAB, 3-ketosteroid 9α-hydroxylase.

Taking all these results into account, PROG catabolism in *C. tardaugens* likely begins with the transformation of PROG into TES-Ac, enabling its entry into the 9,10-seco pathway (20) (Fig. 7). However, we have to consider that PROG can also be dehydrogenated by a KstD to form 1,2-dPROG, an enzyme widely distributed in steroid-degrading bacteria (49–51). KstDs are deeply studied, and mutants in their encoding genes are currently used to accumulate C19 and C21 steroids as pharmaceutical synthons (52–55).

Then, PROG and 1,2-dPROG can undergo a Baeyer-Villiger monooxygenation performed by either BVMO EGO55_13845 or LLM EGO55_13860 to form TES-Ac or BOL-Ac, respectively. The fact that 1,2-dPROG is an intermediate in PROG metabolism does not necessarily imply that the first step of PROG degradation is a C1 dehydrogenation. The C1 dehydrogenation by KtsD enzymes could also occur after the Bayer-Villiger reaction on PROG. In fact, since the accumulated 1,2-dPROG disappears over time, this suggests that the Bayer-Villiger reaction can also occur on 1,2-dPROG, leading to the formation of BOL-Ac instead of TES-Ac.

The transformation of TES-Ac or BOL-Ac into TES or boldenone (BOL) could be carried out by an unknown steryl acetyl hydrolase (56). In this sense, the gene *EGO55_18125* from *C. tardaugens* is annotated as an acetyl hydrolase. The transcriptomic analysis indicates that this gene is neither induced nor repressed, since its TPMs are between 7 and 9 in both PROG and TES conditions. Nevertheless, lipases have also been described to hydrolyze TES esters (57), and we cannot rule out the possibility that lipases or other hydrolases in *C. tardaugens* may play this role.

Once TES or BOL are formed, they enter the TES degradation pathway previously described by Ibero et al. in *C. tardaugens*, where TES and BOL are oxidized into AD and ADD, respectively, by the 3,17β-hydroxysteroid dehydrogenase encoded by the *EGO55_02230* gene (20). AD is dehydrogenated into ADD by a KstD, which is further hydroxylated by a 3-ketosteroid 9α-hydroxylase that opens the steroidal ring A, facilitating its subsequent mineralization via the 9,10-seco pathway.

## DISCUSSION

In spite of the fact that PROG is a critical EDC contaminant, the genes involved in its degradation pathway in bacteria have not been elucidated yet, and the few studies concerning this topic only describe some of the possible intermediates of its degradation pathway. Thus, when Liu et al. studied the aerobic degradation of PROG and norgestrel by bacteria from activated sludge, they detected AD, ADD, 17β-boldenone, TES, 6,7-dehydroprogesterone, and 1,2-dPROG as intermediates in the culture medium (28). They proposed that PROG undergoes different transformations ending up in ADD or 17β-boldenone, which are finally mineralized. Horinouchi et al. also suggested that PROG was transformed somehow into AD and then degraded through the well-described 9,10-seco pathway, but the genes that channel PROG into this degradative pathway were not described (20). Moreover, although a BVMO able to transform PROG into TES-Ac has been isolated in *R. rhodochrous* (34), the PROG catabolic pathway was not described in this microorganism.

Taking advantage of the fact that *C. tardaugens* is able to efficiently use many different steroids including PROG and that we had developed the genetic tools to manipulate this microorganism, we have investigated the fundamental steps involved in the degradation of PROG in bacteria. Because we assumed that most probably the catabolism of PROG and TES was related, we performed a differential transcriptomic analysis of the cells cultured in PROG or TES. This analysis revealed the upregulation of the *pdc* cluster encoding two monooxygenases: a type I BVMO encoded by *EGO55_13845* and an LLM encoded by *EGO55_18860* that transform PROG into TES-Ac through a Bayer-Villiger reaction.

As previously mentioned, BVMOs are a well-known family of proteins that perform a characteristic monooxygenation on a wide range of substrates with a ketone group such as phenylacetone, 2-octanone, cyclobutanone, and different steroids (58, 59). Kołek et al. studied the D-lactonization catalyzed by a BVMO from *Penicillium camemberti* AM83, which transforms steroids such as PROG, TES, and DHEA into testololactone (59). They proposed this fungus as a potential biocatalyst for producing steroidal lactones with novel biologically active applications. The presence of BVMO was postulated as well by Javid et al., showing the bioconversion of progesterone to testololactone by the fungus *Aspergillus sojae* PTCC 5196 through a three-step pathway (17β-acetyl side chain cleavage, 17β-hydroxyl oxidation, and oxygenative lactonization of 17-ketone) (30). The specificity of a BVMO from the bacterium gram-positive *Dietzia* sp. D5 has also been tested and resulted in a wide substrate range including cyclobutanone, phenylacetone, PROG, and even sulfides such as thionisole and ethionamide (58). Moreover, the 3D structure of a BVMO from *R. rhodochrous* transforming PROG into TES-Ac has been solved, leading to the identification of the FAD- and NADP-binding domains and the substrate binding site (33).

On the other hand, LLM enzymes are less studied, likely due to the complexity of their catalysis, which involves a complex of two proteins: a monooxygenase and a reductase. The only known examples are the AbmE2/AbmZ complex from *Streptomyces koyagensis* SCSIO 5802, which catalyzes the inactivation of the antimicrobial abyssomicin 2 to neoabyssomicin B (41), and the 2,5-diketocamphane 1,2-monooxygenases and 3,6-diketocamphane 1,6-monooxygenases, along with their respective reductases, involved in the camphor degradation pathway of *Pseudomonas putida* ATCC 17453 (60, 61).

The functional analyses performed through mutagenesis and subsequent complementation demonstrated that both enzymes are functionally redundant, meaning they can catalyze the same reaction. As a result, both enzymes had to be deleted in order to create a mutant incapable of growing on PROG as its sole carbon and energy source. The functional analyses carried out with the mutant strains showed that PROG can be transformed into 1,2-dPROG most probably due to the dehydrogenation activity of KstD, an enzyme widely distributed in steroid-degrading bacteria (49–51). KstDs are deeply studied, and mutants in their encoding genes are currently used to accumulate C19 and C21 steroids as pharmaceutical synthons (52–55, 62). In a previous work, we demonstrated that the *EGO55_13510* gene encodes a KstD involved in TES degradation in *C. tardaugens* (20). However, other putative *kstD* genes, such as *EGO55_01175* and *EGO55_03150*, are also present within its genome and might play a role in the transformation of PROG into 1,2-dPROG. In this sense, KstDs are known to act on steroid substrates with or without different side chains in C17, since C1 dehydrogenation is a fundamental step in steroid catabolism to facilitate aromatization of the A ring (63). The transcriptomic analysis showed that all *kstD* genes had an FC between −1 and 1 and a TPM between 2 and 150 (Table S1). This suggests that these genes are likely equally expressed under both TES and PROG conditions. The finding that 1,2-dPROG is an intermediate in PROG metabolism does not necessarily indicate that the first step of PROG degradation is C1 dehydrogenation. This is because C1 dehydrogenation by KtsD enzymes could also occur after the Baeyer-Villiger reaction on PROG. In any case, since 1,2-dPROG disappears over time, it suggests that the Baeyer-Villiger reaction can also take place on 1,2-dPROG, leading to the formation of BOL-Ac instead of TES-Ac. The presence of functionally redundant BVMO and LLM enzymes in *C. tardaugens* can be explained by their distinct substrate preferences. Each enzyme may act differentially on steroids other than PROG, such as 1,2-dPROG, contributing to the versatility of the organism’s steroid degradation pathway.

The search for the *pdc* cluster in other bacteria opens up the possibility of identifying microorganisms whose ability to degrade PROG has not yet been described. This is the case of *C. estronivorus*, formerly known as *Altererythrobacter estronivorus*, which is a gram-negative bacterium known to degrade estrogens, although its growth in PROG has not been tested (64). *N. aromaticivorans* is a widely studied strain due to its versatile metabolism, being able to degrade lignin aromatic derivatives and produce valuable compounds. However, its growth in different steroids has not been assessed yet (65–67). The case of *C. testosteroni* is different because it has been demonstrated that it can grow on TES, PROG, and other steroids like cholic acid as carbon and energy source, but the genes involved in PROG degradation had not been identified (68). Remarkably, the *pdc* homologous genes of *N. aromaticivorans* are located in the 487 kb plasmid pNL2, but the BVMO (*Saro_3708*) and TetR-like regulator (*Saro_3709*) are 123 kb separated from the LLM (*Saro_3595*) and the LuxR-like regulator (*Saro_3594*) (Fig. 4). Therefore, this finding suggests that both putative Baeyer-Villiger enzymes have not evolved together in this bacterium and might play different functions. Moreover, both regulators might be involved in controlling the expression of the respective accompanying genes. Why these genes are located contiguously in the genomes of *C. tardaugens* and *C. estronivorus* cannot be easily explained and requires further studies. This comparative analysis provides an interesting observation concerning the role of flavin reductases located close to the *pdc* cluster, since a flavin reductase was not found near the LLM of *N. aromaticivorans* nor the LLM of *N. arvoryzae* (Fig. 4), suggesting that these LLM enzymes could be assisted by other flavin reductases located anywhere else in the genome.

## MATERIALS AND METHODS

### Chemicals

PROG, 21-OHPROG, PREG, TES, TES-Ac, TES-Cyp, AD, ADD, DHEA, E2, sodium cholate, sodium deoxycholate, chloroform, *n*-hexane, ethyl acetate, and acetonitrile were purchased from Merck KGaA Sigma (Germany). Randomly methylated β-cyclodextrin (CDX) was purchased from Cyclolab R&D Ltd. (Hungary). 1,2-dPROG was purchased from Toronto Research Chemicals (Canada).

### Strains and growth media

All bacterial strains, plasmids, and primers used in this study are listed in Table 1. *C. tardaugens* NBRC 16725 (formerly known as *N. tardaugens*) was obtained from the Leibniz-Institut DSMZ-type culture collection. This strain and all its mutants were cultured as described (20). *C. tardaugens* was cultured in NB (Difco) as rich medium at 30°C and 200 rpm in an orbital shaker, while the minimal medium used was M63 (KH_2_PO_4_ [136 g/L], (NH_4_)_2_SO_4_ [20 g/L], FeSO_4_·7H_2_O [5 mg/L], pH 7.0) supplemented with 0.39 mM CaCl_2_, 1 mM MgSO_4_, and the appropriate carbon source concentration. Steroids stock solutions were prepared in phosphate-buffered saline (PBS) buffer (per liter, 8 g NaCl, 0.2 g KCl, 1.44 g Na_2_HPO_4_, and 0.24 g KH_2_PO_4_; pH 6.8) with 70 mM CDX, and the final concentration in the culture was 13.33 mM CDX and 1.71 mM and 1.89 mM for PROG and TES, respectively. *Escherichia coli* DH10B, *E. coli* BL21(DE3), and *E. coli* HB101 strains were grown in lysogeny broth (LB) medium at 37°C and 200 rpm in an orbital shaker. When needed, rifampicin (50 µg/mL) and kanamycin (50 µg/mL or 10 µg/mL) were added for *E. coli* and *C. tardaugens* strains, respectively.

### RNA extraction

Total RNA extraction of *C. tardaugens* was performed as described (20). Cells from three biological replicates were first cultured on minimal medium with PROG or TES as carbon sources until they reached the mid-exponential phase (OD_600_ = 0.6), when they were harvested and stored at −80°C. Pellets were then thawed and lysed in 400 µL TE buffer (10 mM Tris-HCl, 1 mM EDTA, pH 7.5) with lysozyme (50 mg/mL). Three freezing–thawing cycles were performed before using the High Pure Isolation Kit (Roche, Switzerland), followed by DNA-free DNA Removal Kit (Invitrogen, UK) treatment to obtain pure RNA. Purity and concentration were measured in a ND1000 spectrophotometer (Nanodrop Technologies, USA), while RNA integrity was checked in an Agilent Technologies 2100 Bioanalyzer (USA).

### Transcriptomic analysis (RNA-seq)

Transcriptome sequencing was done by Macrogen NGS Service, using an Illumina TruSeq RNA library with 6GB/sample sequencing coverage, obtaining fragments of 151 bp paired-end reads. Bioinformatics analyses were performed by the Bioinformatics and Biostatistics Service of the Center for Biological Research Margarita Salas (CIBMS-CSIC). Raw reads data quality was checked using FastQC, and then they were trimmed and cleaned with Trimmomatic 0.39 (69). After filtration, 55,475,633 trimmed reads were obtained, and 45,845,222 million high-quality clean reads were mapped to the genome of *C. tardaugens* (accession number CP034179) using Bowtie2 2.4.2 (70). Expression quantification was done using HTSeq-count 0.13.5 (71), and differential gene expression analysis was performed using DESeq2 1.32.0 from the R software v.4.3.2. (R: The R Project for Statistical Computing; https://www.r-project.org/). Genes presenting an FC > 2 or FC < -2 and false discovery rate < 0.05 (represented as *padj* in Table S1) were considered as differentially expressed. In the dissimilarity matrix shown in the heatmap, the color scale represents the log2 of median of ratios normalized counts plus one, and the dendrogram was obtained with the Euclidean distance between genes and the complete linkage hierarchical method for clustering. The figures were done with the package ComplexHeatmap (72) in the R software v.4.3.2, indicated before.

### Construction of *C. tardaugens* knockout strains and their complementation

Knockout strains of *C. tardaugens* were constructed by double homologous recombination as described (20). Briefly, an upstream and a downstream region of about 700 bp flanking the gene to delete were amplified and cloned using *Bsa*I in the suicide vector pK18msg (Table 1), a variant of pk18*mobsacB* where the multiple cloning site (MCS) was substituted by type II restriction enzymes kindly provided by Godoy et al. (46). The recombinant vector was transformed into *E. coli* DH10B competent cells as described (73) and checked by PCR and by sequencing of the cloned region. Then, *C. tardaugens* rifampicin resistant was transformed with the suicidal plasmid by triparental conjugation using *E. coli* HB101 (pRK600) as helper and *E. coli* DH10B, harboring the corresponding vector, as donor (47, 74). The result of the conjugation was plated on NB agar with 10 µg/mL kanamycin and 50 µg/mL rifampicin. Candidates were checked by colony PCR to ensure that the suicide vector is inserted in the genome. Then, they were cultured to stationary phase and plated on NB agar with 5% sucrose. Only the clones that were able to grow in the presence of sucrose and sensitive to kanamycin were checked by PCR in order to confirm the complete deletion of the gene after the second recombination event.

*C. tardaugens* mutants were complemented with plasmids belonging to the pSEVA collection (pSEVA23) by triparental conjugation as described above. The genes *EGO_13845* and *EGO55_13860,* previously deleted, were amplified using the primers indicated in Table 1, cloned in *Xba*I-*Sph*I and *Hin*dIII-*Spe*I sites, respectively, and expressed under the *P_lexA_* constitutive promoter expression (48).

### Cloning and expression of *EGO55_13845* and *EGO55_13860*

*EGO55_13845* and *EGO55_13860* genes, encoding a BVMO and LLM, respectively, were amplified with primers described in Table 1 and cloned separately in the pET-29a(+) vector using the Gibson Assembly Cloning Kit (New England Biolabs) following the manufacturer instructions. The resulting pET-BVMO and pET-LLM plasmids were then electroporated in *E. coli* BL21(DE3) electrocompetent cells using a Gene Pulser (Bio-Rad, USA) (75). Transformed strains were cultured in 50 mL of LB with kanamycin (50 µg/mL) up to an OD_600_ of 0.5–0.8 at 37°C and 200 rpm. Then, isopropyl β-D-1-thiogalactopyranoside (IPTG) was added to a final concentration of 0.2 mM to induce gene expression. Cultures were then kept at 30°C for 3 h. Next, cells were harvested and washed twice with 0.85% saline solution and finally resuspended in 50 mM phosphate buffer (pH 8.0). A Branson sonicator (Germany) was used to lyse the cells with three cycles of 10 s at maximum power and 30 s cooling on ice. Crude extract was centrifuged at 13,000 × *g* in a microfuge Sigma 1-15K (Germany) for 15 min at 4°C to obtain the soluble fraction. Protein concentration was calculated with the Bradford method (76), and overproduction of proteins was visualized by SDS-polyacrylamide gel electrophoresis (Fig. S6).

### Enzymatic assay of BVMO and LLM activities

BVMO activity was assayed in a Shimadzu UV-1900i spectrophotometer (Japan) by measuring the decrease in absorbance of NADPH at 340 nm (ε_NADPH_ = 6.22 cm^−1^ mM^−1^) when the substrate was added. The enzymatic reaction was done in quartz cuvettes (HELLMA Analytics, 10 mm) containing 0.6 mL of 2 mg/mL of crude extract in 50 mM sodium phosphate buffer (pH 8.0) at 25°C. Substrate stocks were prepared in methanol at 50 mM to obtain a final concentration of 0.5 mM, and the reaction started when 0.2 mM NADPH was added. In this case, 1 unit of specific activity (U/mg) was defined as the number of nanomoles of NADPH consumed in 1 min per milligram of protein.

The LLM enzymatic assays were performed as follows: 0.6 mL of 4 mg/mL of crude extract in 50 mM sodium phosphate buffer (pH 7.5) was added to a 4 mL Clear Vial (Supelco, USA) with magnetic agitation at 25°C. Substrate stocks were prepared as described before and added at a final concentration of 0.5 mM, along with 20 µM of FMN. Reaction started when 5 mM NADH was added. A total of 200 µL samples was taken at 0, 5, and 30 min and mixed with 500 µL of chloroform to stop the reaction. Steroids were extracted and analyzed as described below. Since product standards were not available, 1 unit of specific activity (U/mg) was defined as the number of nanomoles of substrate consumed in 1 min per milligram of protein.

To determine the enzymatic activities in protein crude extracts of *C. tardaugens*, the WT and mutant strains were grown in 12.5 mL of NB in the presence of 1.71 mM PROG to ensure *EGO55_13845* and *EGO55_13860* induction. When the culture reached the mid-exponential phase (OD_600_ = 1), cells were harvested and washed twice with 0.85% saline solution. Finally, cells were resuspended in 50 mM phosphate buffer (pH 8.0) and sonicated as described before. Crude extract was centrifuged at 13,000 × *g* in a microfuge Sigma 1-15K (Germany) for 15 min at 4°C to obtain the soluble fraction. Thereafter, 0.6 mL of protein crude extracts was tested in a 4 mL Clear Vial (Supelco, USA) at a final concentration of 4 mg/mL. PROG was added at a final concentration of 0.5 mM with 20 µM FMN in 50 mM sodium phosphate buffer (pH 7.5). The reaction conditions were maintained at 25°C with magnetic agitation. Reaction started when 2 mM of NADPH or NADH was added. Samples of 200 µL were taken at 0 and 30 min of reaction time for their analyses.

### Organic phase extraction and chromatographic analysis

The steroidal compounds from the culture media and from the enzymatic assays were extracted by organic solvent extraction with chloroform as described before (20). Briefly, two volumes of chloroform were added to the sample, vortexed, and then the organic phase was extracted, dried, and resuspended in 150 µL of acetonitrile. Extracted steroids were detected and identified using high-efficiency liquid chromatography coupled to a photodiode array detector and mass spectroscopy (HPLC-MS) as described in Hernández-Fernández et al. (54). Samples were analyzed in a 1260 Infinity II (Agilent, USA) with InfinityLab LC/MSD XT (Agilent, USA), with automatic injector, diode array detector, and ion trap with electrospray. For the separation method, a Poroshell 120 EC-C18 (4 µm; 4.6 × 100 mm) (Agilent, USA) column was used, with a flow rate of 1 mL/min starting with 70% of solvent A (H_2_O + 0.1% formic acid) and 30% of solvent B (acetonitrile + 0.1% formic acid) for 5 min, followed by an increase of solvent B to 55% for 20 min and then reduced back to 30% for 2 min. This rate was finally maintained for 3 min. The elution was monitored at 195, 210, 230, 250, and 275 nm, and the steroid compounds obtained were identified, thanks to the commercial standards comparing mass spectra and retention time.

To identify the steroid compounds, a GC-MS analysis was performed as described (55). Previously to the steroid extraction, 25 µL of cholestenone 10 mM was added to each sample as an internal standard. Once extracted, trimethylsilyl ether derivatives were formed by adding 50 µL of N,O-bis(trimethylsilyl)trifluoroacetamide with trimethylchlorosilane (Merck, Germany) and 50 µL of pyridine. This solution was then heated at 80°C for 20 min. An Agilent 7890A gas chromatograph coupled to an Agilent 5975 C mass detector (Agilent Technologies, USA) was used for the GC-MS analysis. Mass spectra were recorded in electron impact mode at 70 eV within an *m*/*z* range set between 50 and 550. A 30 m × 0.25 mm i.d. capillary column (0.25 µm film thickness) HP-5MS (5% diphenyl, 95% dimethylpolysiloxane) (Agilent Technologies, USA) was used with the chromatograph, and working conditions were a split ratio of 20:1, 320°C for the injector temperature. Then the column temperature was set at 240°C for 3 min and finally heated to 320°C at 5°C min^−1^. In order to identify the compounds obtained, mass spectra and retention data were compared to those observed in the commercial standards and to those present in the NIST Mass Spectral Database (NIST 2011).

## Data Availability

Raw read data obtained from the three replicates of the transcriptome of the strain grown on PROG and TES have been deposited in the Sequence Read Archive (SRA) database of the National Center for Biotechnology Information (NCBI) under accession numbers SRR31340002 and SRR31340003 (BiopProject PRJNA1185793), respectively. The original contributions presented in this study are included in this article and supplemental material; further inquiries can be directed to the corresponding author.

## SUPPLEMENTAL MATERIAL

Supplemental figures mbio.01077-25-s0001.pdf Figures S1 to S9.

Table S1 mbio.01077-25-s0002.xlsx Transcriptomic analysis of *C. tardaugens* in progesterone versus testosterone.

ASM does not own the copyrights to Supplemental Material that may be linked to, or accessed through, an article. The authors have granted ASM a non-exclusive, world-wide license to publish the Supplemental Material files. Please contact the corresponding author directly for reuse.
