# Supplementary material for: Unraveling the key drivers of bacterial progesterone degradation
Source: mBio. 2025 May 30;16(7):e01077-25. doi: 10.1128/mbio.01077-25 (PMC12239570; doi:10.1128/mbio.01077-25)
Supplement: Supplemental figures — Figures S1 to S9. [file mbio.01077-25-s0001.pdf]

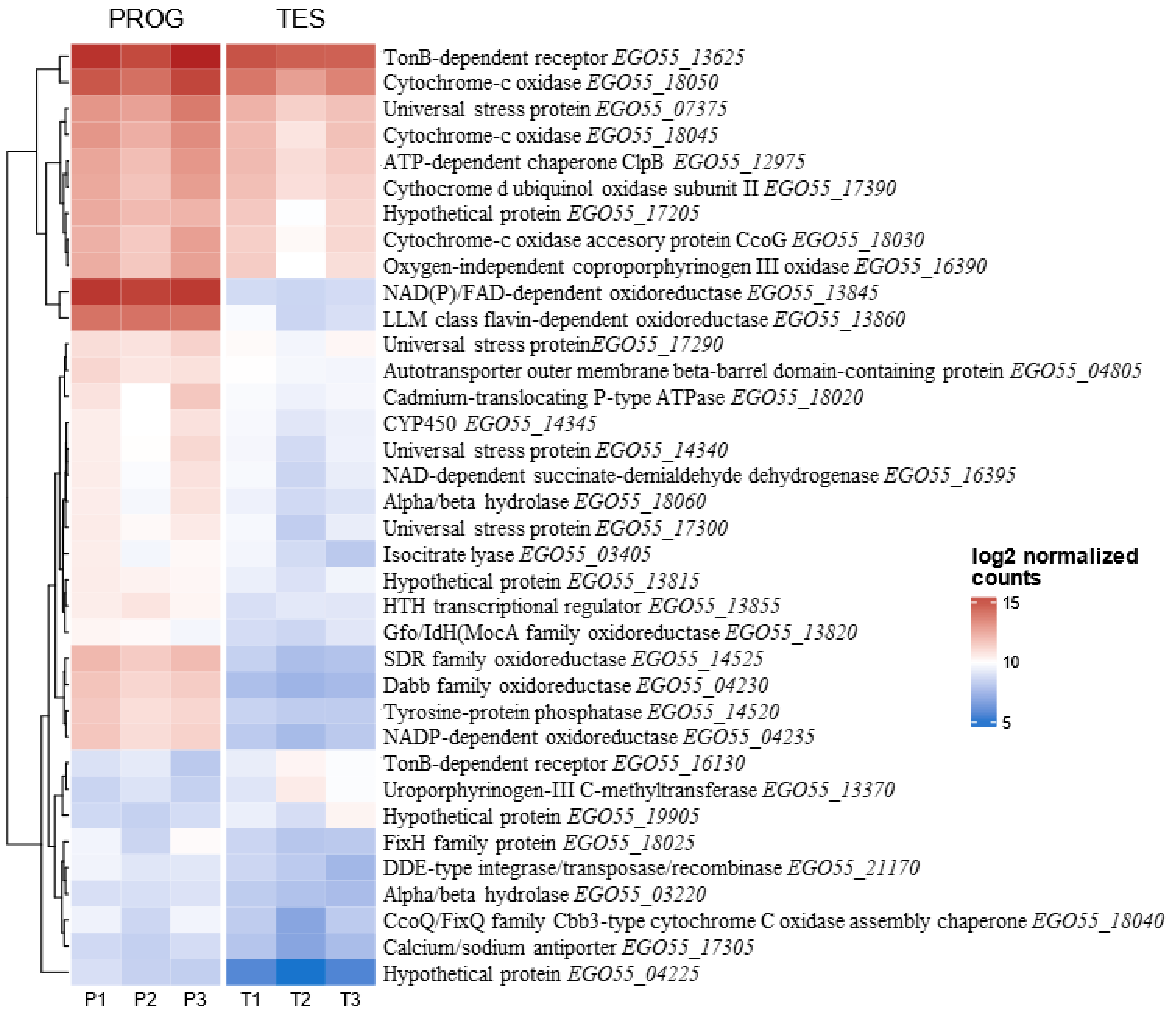

Figure S1. Heatmap of the 36 DEGs according to a FC>|2| and an FDR<0.05. Each DEG is followed with its annotation and locus tag.

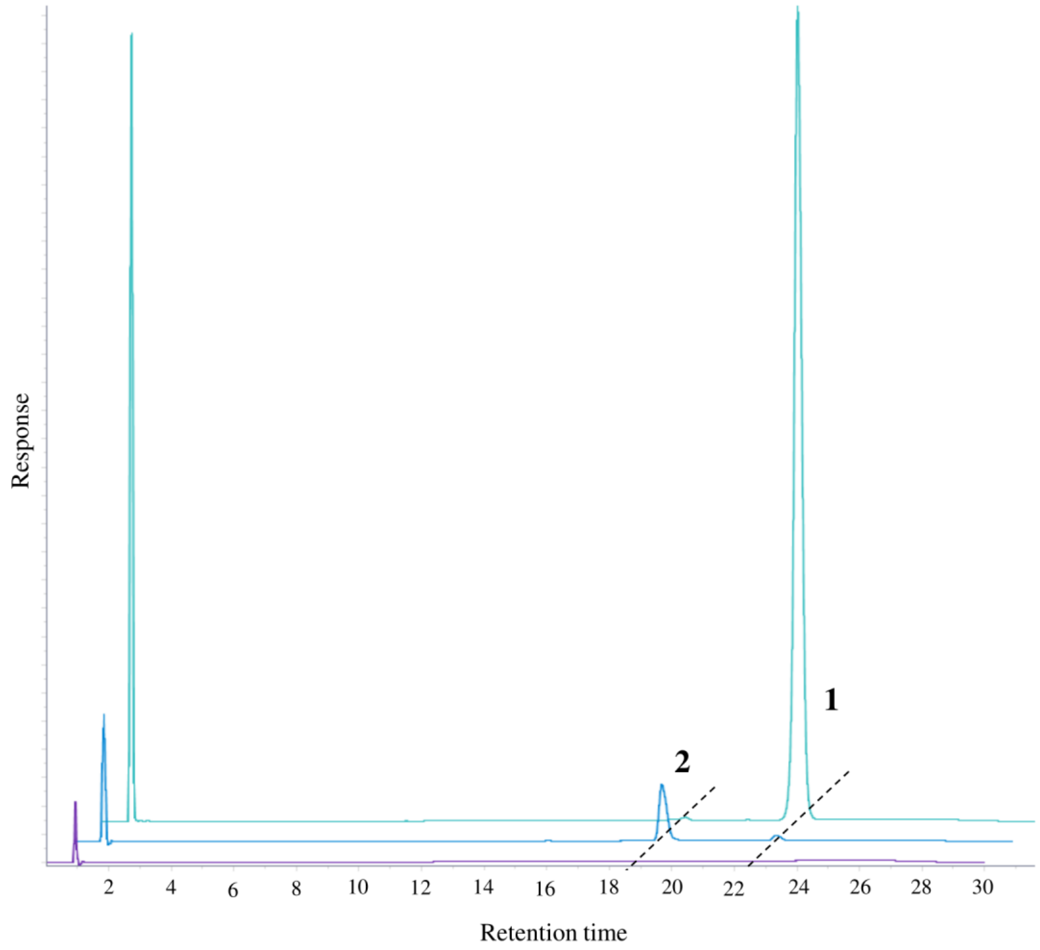

Figure S2. HPLC analysis of the organic phase extracted from *C. tardaugens* ΔLLM strain grown with progesterone after 0 h (turquoise), 30 h (blue) and 39 h (purple). Peak 1 correspond to progesterone and peak 2 correspond to 1,2-dehydroprogesterone.

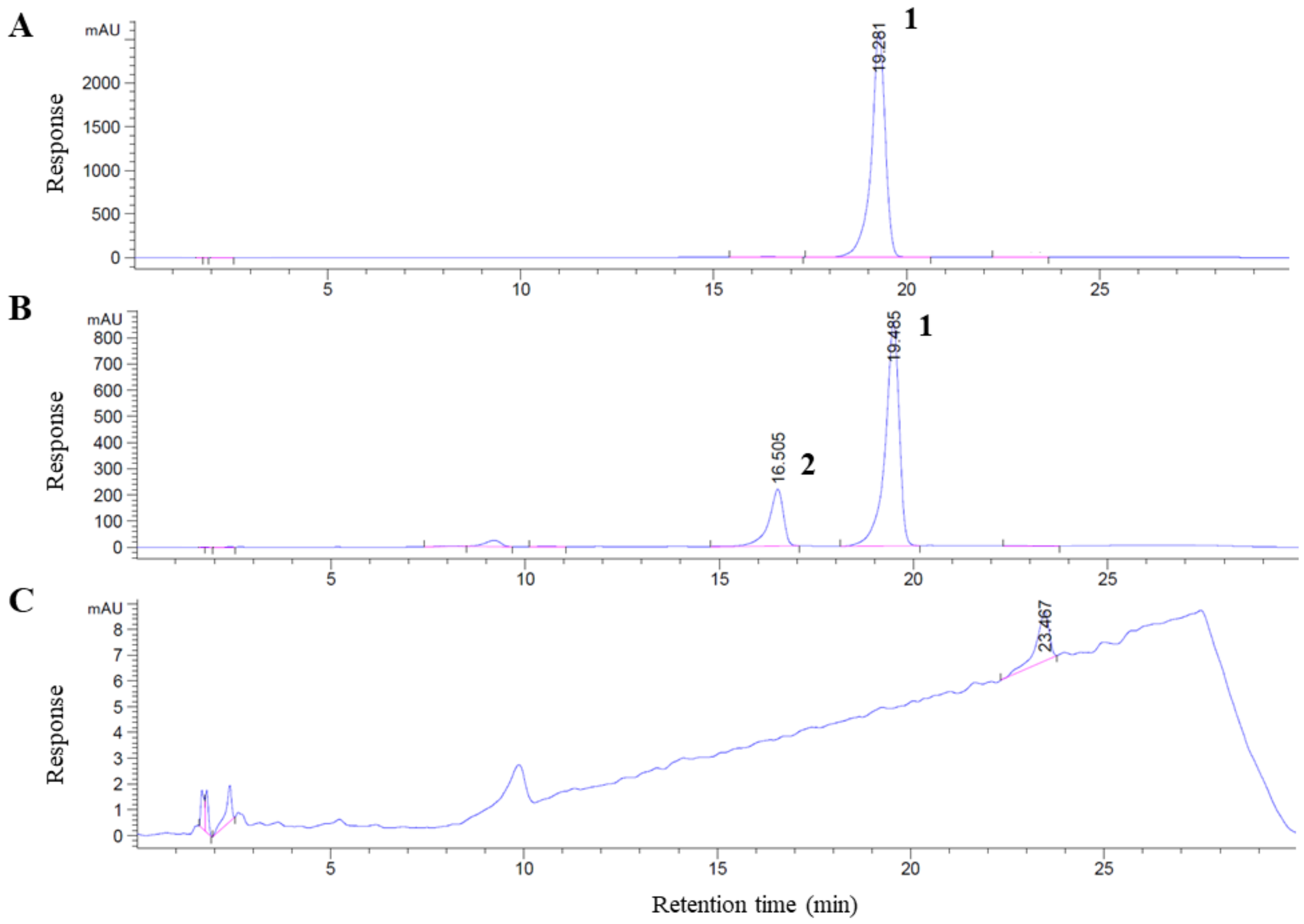

Figure S3. HPLC analysis of the organic phase extracted from *C. tardaugens* ΔBVMO strain grown with progesterone after 0 h (A), 18 h (B) and 24 h (C). Peak 1 correspond to progesterone and peak 2 correspond to 1,2-dehydroprogesterone.

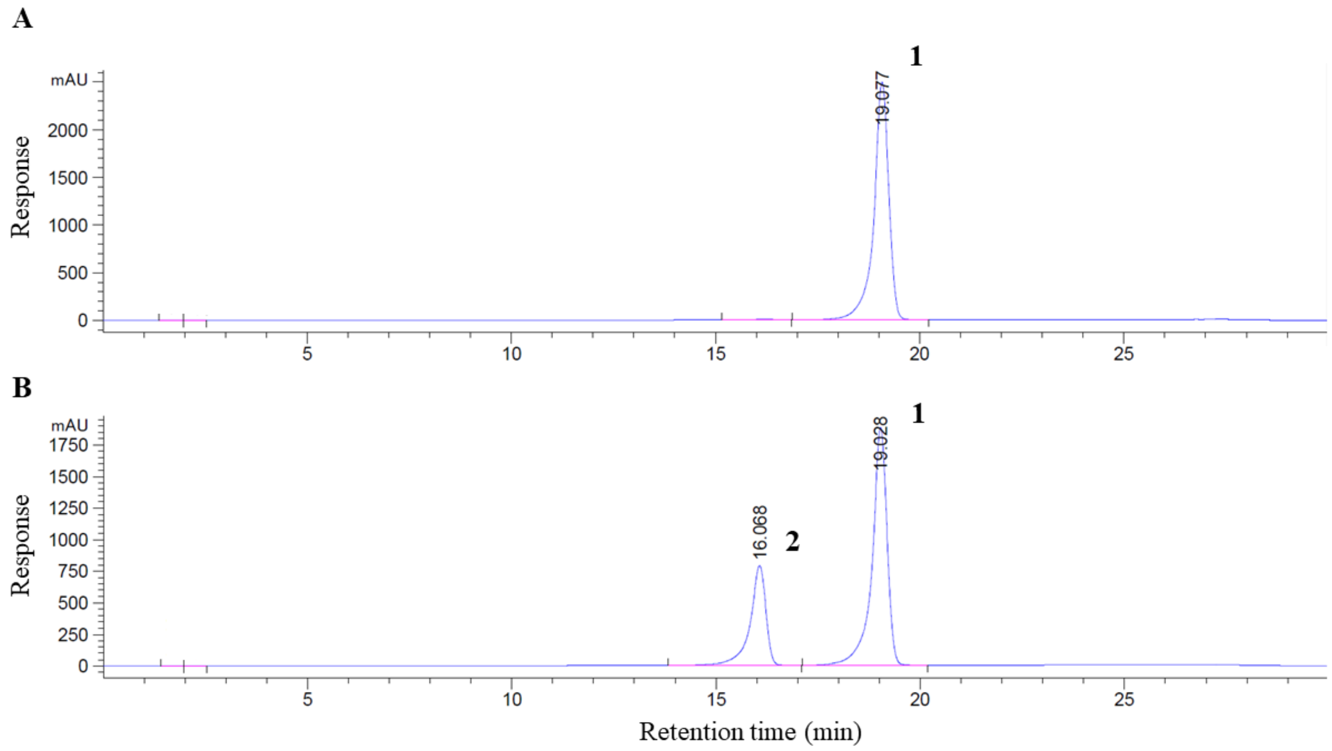

Figure S4. HPLC analysis of the organic phase extracted from *C. tardaugens* ΔLLM-BVMO strain grown with progesterone after 0 h (A) and 96 h (B). Peak 1 correspond to progesterone and peak 2 correspond to 1,2-dehydroprogesterone.

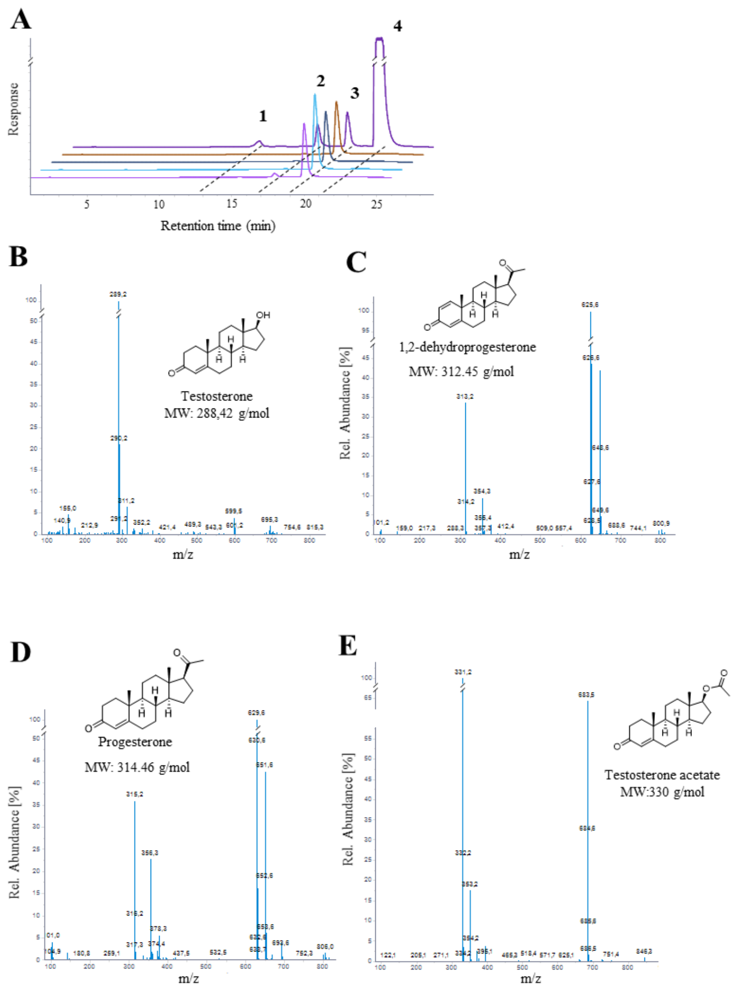

Figure S5. (A) HPLC-MS analysis of the organic phase extracted from the enzymatic reaction of progesterone with crude extracts from *C. tardaugens* WT 0h (brown) and 30 min (dark gray) compared to *C. tardaugens* ΔLLM-BVMO 0h (light blue) and 30 min (lilac). Commercial TES (peak 1), 1,2-dPROG (peak 2), PROG (peak 3) and TES-Ac (peak 4) are represented in purple. (B) Mass spectra corresponding to peak 1. (C) Mass spectra corresponding to peak 2. (D) Mass spectra corresponding to peak 3. (E) Mass spectra corresponding to peak 4.

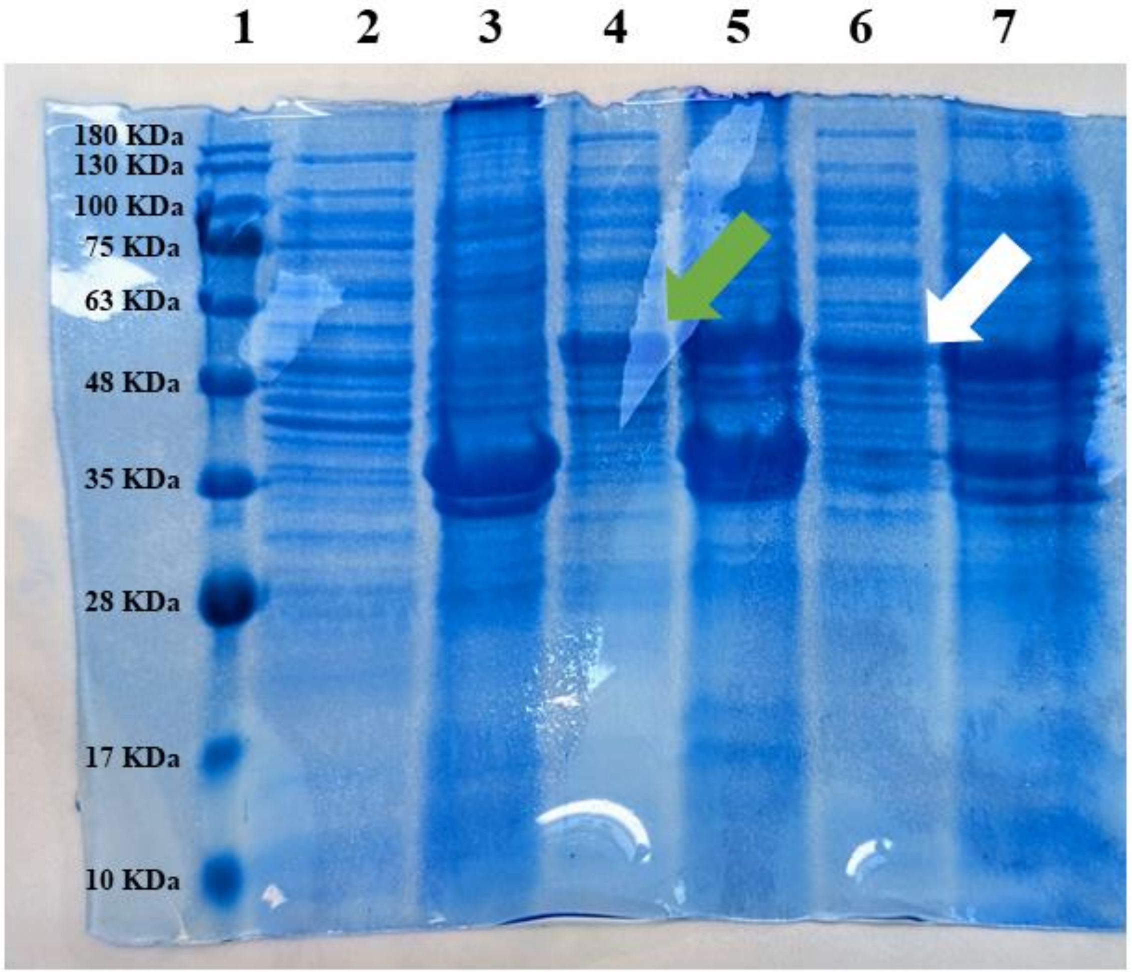

Figure S6. SDS-PAGE with crude extracts of *E. coli* BL21 (pET-29a(+)) (lane 2: soluble fraction; lane 3: insoluble fraction), *E. coli* BL21 (pET-29a(+)BVMO) (lane 4: soluble fraction; lane 5: insoluble fraction) and *E. coli* BL21 (pET-29a(+)LLM) (lane 6: soluble fraction; lane 7: insoluble fraction). The molecular weight marker is present in lane 1. BVMO protein is marked with a green arrow while LLM protein is marked with a white arrow.

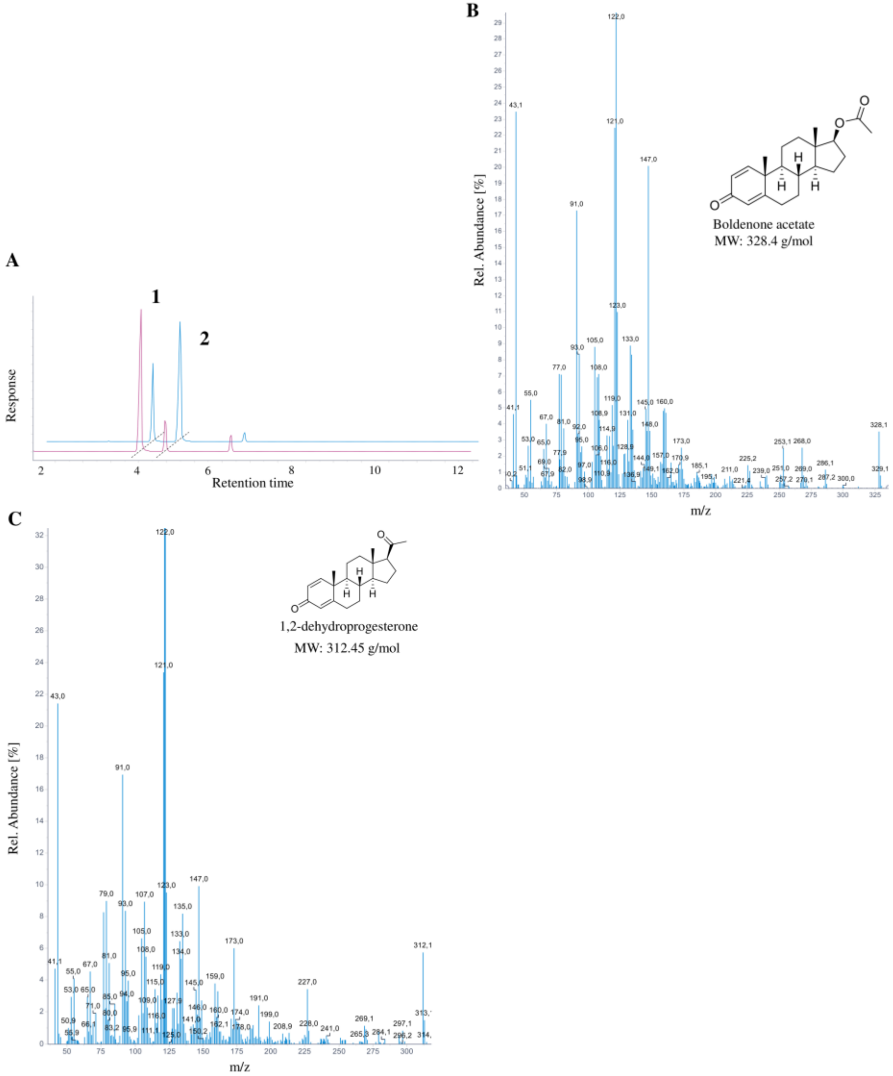

Figure S7. (A) GC-MS analysis of the enzymatic assay after 30 min containing BVMO (blue) or LLM (burgundy) with 1,2-dPROG (peak 2) yielding BOL-AC (peak 1). (B) Mass spectra corresponding to peak 1. (C) Mass spectra corresponding to peak 2.

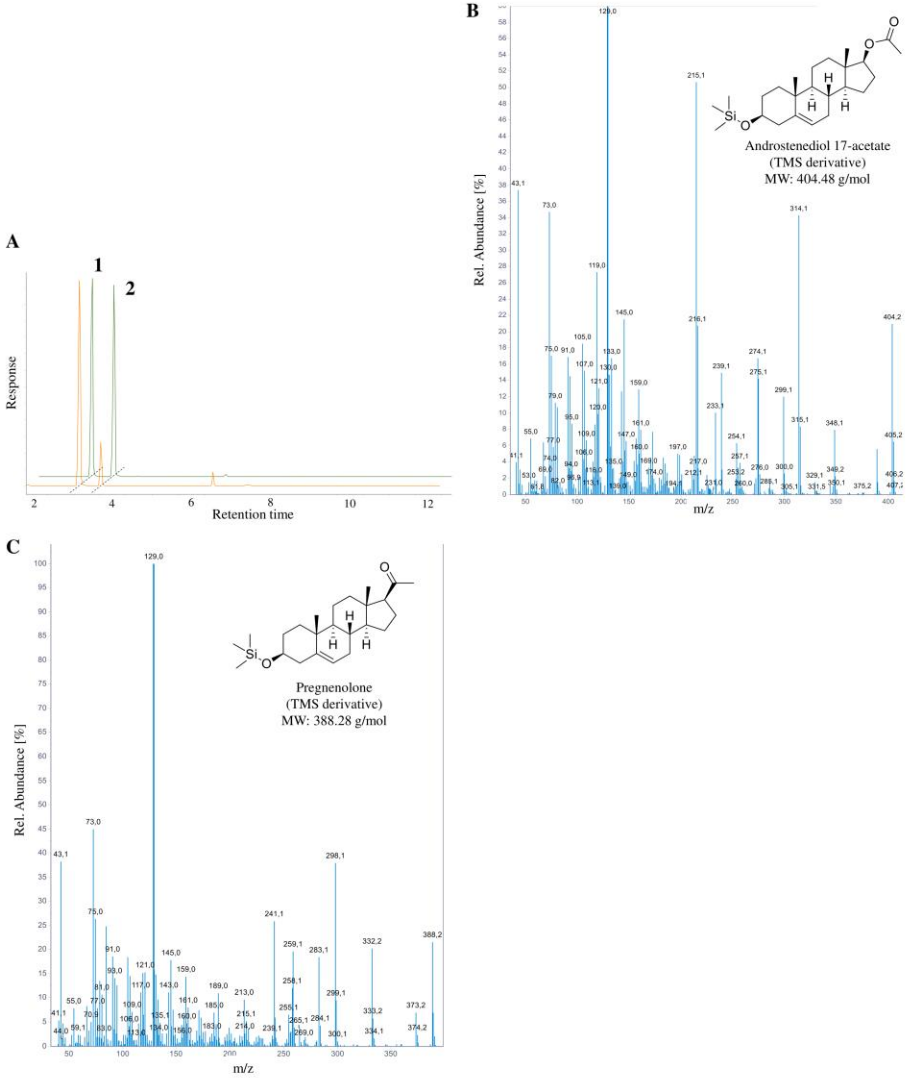

Figure S8. (A) GC-MS analysis of the enzymatic assay after 30 min containing BVMO (green) or LLM (yellow) with PREG (peak 2) yielding DHEA-Ac. (B) Mass spectra corresponding to peak 1. (C) Mass spectra corresponding to peak 2.

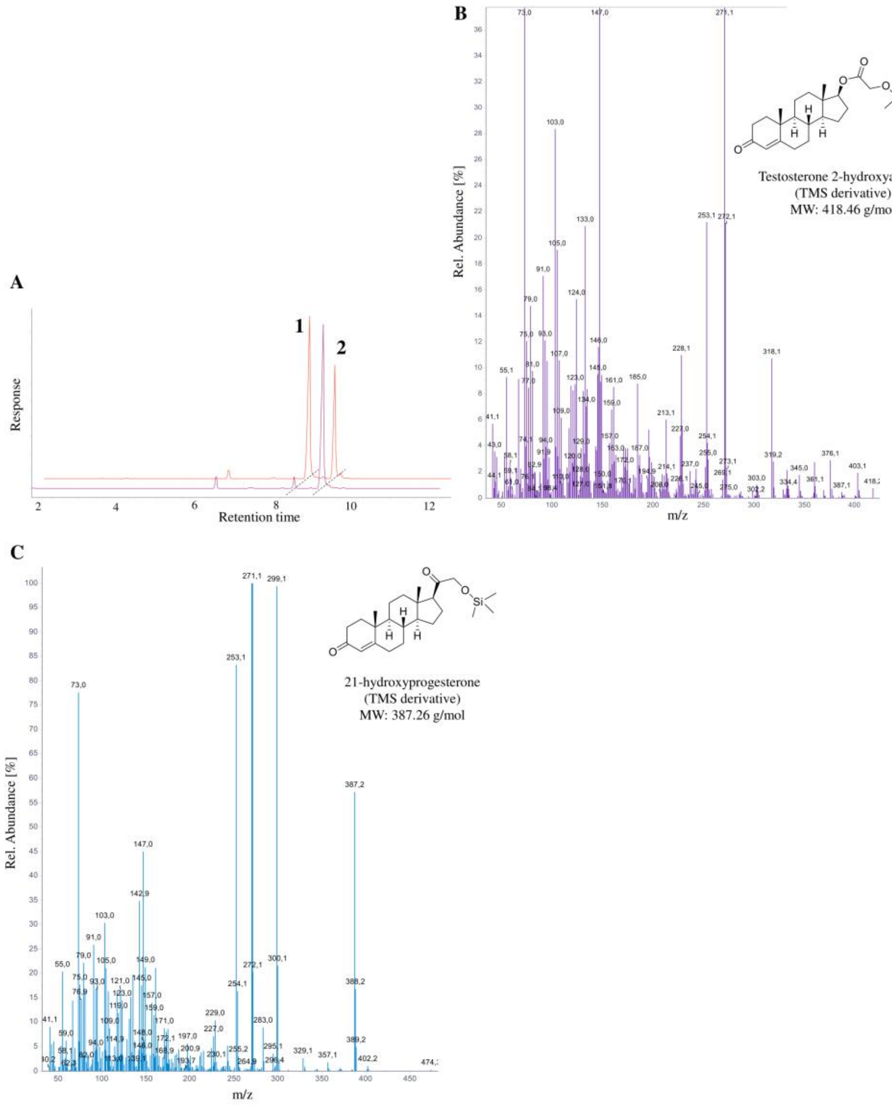

Figure S9. (A) GC-MS analysis of the enzymatic assay after 30 min containing BVMO (orange) or LLM (burgundy) with 21-OHPROG (peak 1) yielding testosterone 2-hydroxyacetate. (B) Mass spectra corresponding to peak 2. (C) Mass spectra corresponding to peak 1.
